# Gut microbiota-derived inosine from dietary barley leaf supplementation attenuates colitis through PPARγ signaling activation

**DOI:** 10.1186/s40168-021-01028-7

**Published:** 2021-04-05

**Authors:** Daotong Li, Yu Feng, Meiling Tian, Junfu Ji, Xiaosong Hu, Fang Chen

**Affiliations:** 1 College of Food Science and Nutritional Engineering, National Engineering Research Center for Fruit and Vegetable Processing, Key Laboratory of Fruits and Vegetables Processing, Ministry of Agriculture; Engineering Research Centre for Fruits and Vegetables Processing, Ministry of Education, China Agricultural University, No.17, Qinghua East Road, Haidian District, Beijing, 100083 China; 2 Department of Anatomy, Histology and Embryology, Health Science Center, Peking University, Beijing, China

**Keywords:** Barley leaf, Colitis, Gut microbiota, Purine metabolite, Mucosal barrier, PPARγ, Inosine

## Abstract

**Background:**

Ulcerative colitis is a type of chronic inflammatory bowel disease closely associated with gut microbiota dysbiosis and intestinal homeostasis dysregulation. Barley leaf (BL) has a long history of use in Traditional Chinese Medicine with potential health-promoting effects on intestinal functions. However, its mechanism of action is not yet clear. Here, we explore the potential modulating roles of gut microbial metabolites of BL to protect against colitis and elucidate the underlying molecular mechanisms.

**Results:**

Using 16S rRNA gene-based microbiota analysis, we first found that dietary supplementation of BL ameliorated dextran sulfate sodium (DSS)-induced gut microbiota dysbiosis. The mechanisms by which BL protected against DSS-induced colitis were resulted from improved intestinal mucosal barrier functions via the activation of peroxisome proliferator-activated receptor (PPAR)γ signaling. In addition, metabolomic profiling analysis showed that the gut microbiota modulated BL-induced metabolic reprograming in the colonic tissues particularly by the enhancement of glycolysis process. Notably, dietary BL supplementation resulted in the enrichment of microbiota-derived purine metabolite inosine, which could activate PPARγ signaling in human colon epithelial cells. Furthermore, exogenous treatment of inosine reproduced similar protective effects as BL to protect against DSS-induced colitis through improving adenosine 2A receptor (A_2A_R)/PPARγ-dependent mucosal barrier functions.

**Conclusions:**

Overall, our findings suggest that the gut microbiota-inosine-A_2A_R/PPARγ axis plays an important role in the maintenance of intestinal homeostasis, which may represent a novel approach for colitis prevention via manipulation of the gut microbial purine metabolite.

Video Abstract

**Supplementary Information:**

The online version contains supplementary material available at 10.1186/s40168-021-01028-7.

## Background

Inflammatory bowel disease is a chronic inflammatory disease of the intestine that includes ulcerative colitis and Crohn’s disease [1]. Ulcerative colitis is characterized by the presence of discontinuous lesions that mainly affect the mucosal layer in the cecum and colon [2]. Although it is highly prevalent in the developed countries, recent studies have shown that it is becoming more frequent in developing countries and this disease has emerged as a worldwide public health challenge [3, 4]. Although the etiology of ulcerative colitis is still not fully understood, multiple genetic and environmental factors have been reported to be involved in the pathogenesis of ulcerative colitis [5]. Patients with ulcerative colitis display decreased expression of peroxisome proliferator-activated receptor (PPAR)γ in the colonic epithelium, which may be an important factor for the cause of intestinal dysfunction and chronic inflammation [6]. Clinically, PPARγ is also a crucial pharmacological target for anti-inflammatory drugs such as 5-aminosalicylic acid and corticosteroids [7]. However, most of the current drug-based interventions lack specificity and may have adverse effects [8]. There is therefore an unmet need for developing new therapeutic strategies for the prevention and treatment of colitis.

The gut microbiota, which contains trillions of metabolically active microbes, is an important environmental factor affecting host physiology and shaped by diet [9]. The intestinal mucus and epithelial cell layer is the first line of defense limiting the translocation of potentially harmful antigens [10]. Interestingly, germ-free and conventionally raised mice exhibited different properties of colonic mucus layer, which were also observed in mice with different gut microbiota composition. Notably, the different properties of colonic mucus layers could be transmissible to germ-free mice through gut microbiota transplantation [11, 12]. Moreover, another study showed that transplantation of the gut microbiota from patients with irritable bowel syndrome could lead to alterations of intestinal motility and barrier functions in germ-free mice [13]. These findings suggest that the gut microbiota may serve as a pivotal mediator in the maintenance of intestinal homeostasis and targeting the microbiota could be an effective therapeutic approach for preventing and curing colitis [14].

It has been proposed that the effects of the gut microbiota on the host physiological functions partially depend on small molecules derived from host and microbiota co-metabolism, such as short-chain fatty acids, bile acids, and indole derivatives, which are greatly influenced by dietary nutrients [15–20]. Inosine, a primary metabolite of adenosine, is an important intracellular purine nucleoside. By acting as the functional agonist of the adenosine receptors, inosine is involved in the regulation of many physiological and pathophysiological processes [21]. It should be noted that inosine is also an important metabolite that is produced by gut bacterial species. Mager et al. showed that gut microbiome-derived metabolite inosine could enhance the effects of checkpoint blockade immunotherapy via activation of antitumor T cells [22].

Barley leaf (BL), the young grass of the crop barley (*Hordeum vulgare* L.), is the primary component of a popular green-colored functional drink in Asian countries [23]. The antioxidative properties of BL have been reported in previous studies [24, 25]. Moreover, as an herbal-based component of Traditional Chinese Medicine, it is historically recorded to have potential health-promoting properties on intestinal functions. However, whether the gut microbiota-derived metabolites play a part in modulating the beneficial effects of BL on intestinal functions and the underlying mechanism have not been elucidated.

In this study with a mouse colitis model, we demonstrated that BL significantly mitigated disease severity and microbiota dysbiosis. We revealed that the underlying mechanisms were related to the improved mucosal barrier functions via peroxisome proliferator-activated receptor (PPAR)γ signaling activation. Antibiotic treatment further demonstrated that the gut microbiota was involved in BL-induced metabolic reprogramming in colonic tissues. Additionally, we identified a gut microbiota-derived purine metabolite inosine, which mediated the beneficial effects of BL on intestinal functions through adenosine 2A receptor (A_2A_R)/PPARγ signaling. Thus, our data provide new insights into the underlying mechanisms of BL in colitis protection and highlighted the potential application of BL and inosine for the prevention of ulcerative colitis.

## Results

### BL ameliorates colitis symptoms and gut microbiota dysbiosis

**Fig. 1:**
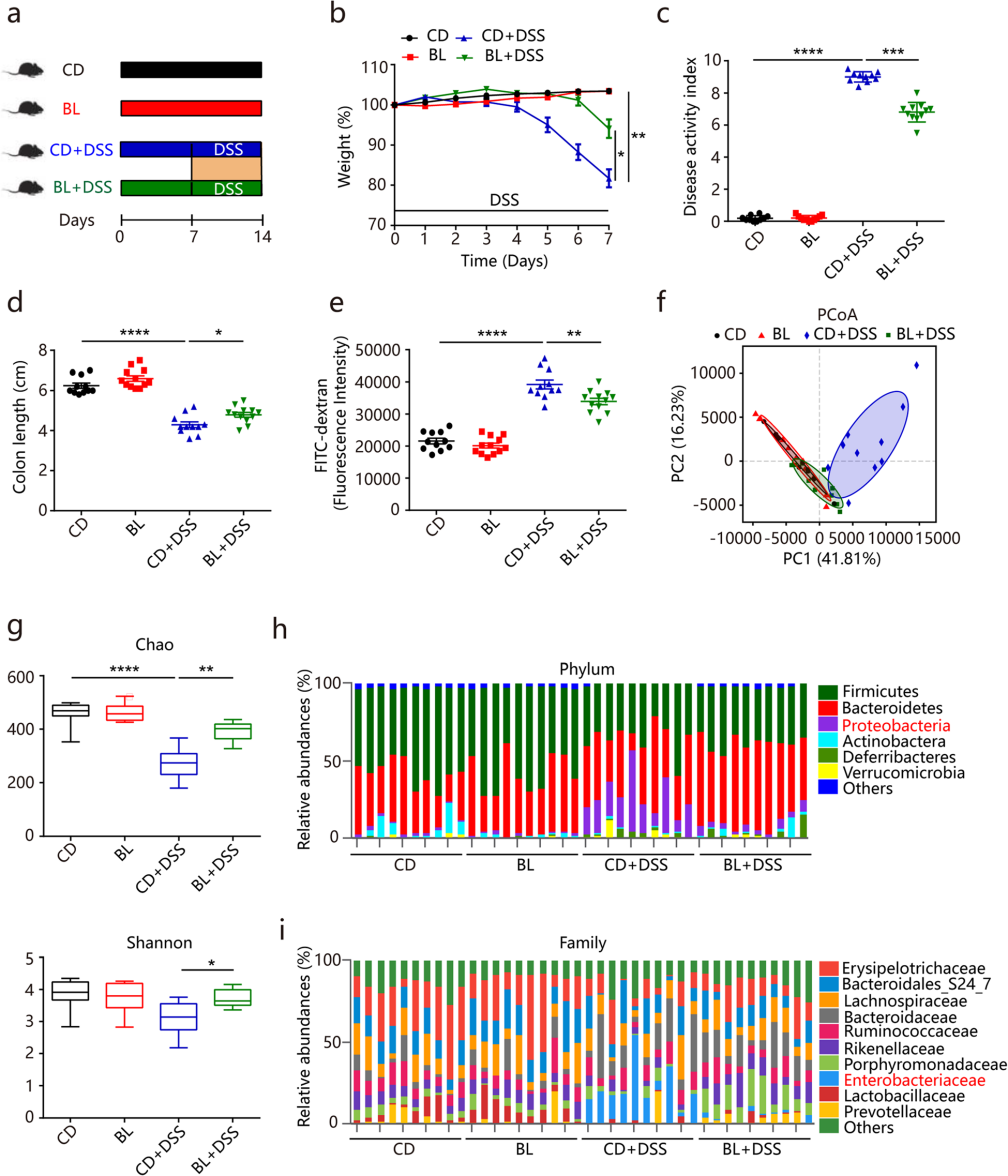
Barley leaf (BL) attenuates dextran sulfate sodium (DSS)-induced colitis and gut microbiota dysbiosis. **a**–**i** Mice were fed with a standard chow diet (CD) or an isocaloric BL-supplemented diet for two weeks. Colitis was induced by administering 2.5% DSS dissolved in drinking water for 7 days. **a** Study design of in vivo mouse experiment. **b** Percentage body weight change, **c** diseases activity scores, **d** colon lengths, and **e** intestinal permeability of the CD- and BL-fed mice with or without DSS treatment (*n* = 10–12). **f** Weighted UniFrac Principal coordinate analysis (PCoA) plot of the gut microbiota composition at the operational taxonomic unit (OTU) level from different mouse groups (*n* = 10). **g** Alpha diversity analysis of gut bacterial richness (Chao1 index) and diversity (Shannon index) from different mouse groups (*n* = 10). **h** Taxonomic distributions of gut bacterial composition at the phylum level (*n* = 10). **i** Taxonomic distributions of gut bacterial composition at the family level (*n* = 10). Data are pooled from three independent experiments (a-e). Data are mean ± SEM. *P < 0.05, **P < 0.01, ***P < 0.001 and ****P < 0.0001. For body weight change, a repeated measure two-way analysis of variance (ANOVA) was performed and the rest of the statistics was performed with one-way ANOVA followed by Tukey’s multiple comparisons test

In this study, the beneficial impacts of BL on intestinal functions were examined in a dextran sodium sulfate (DSS)-induced colitis mouse model, which is a widely used model exhibiting several characteristics resembling human ulcerative colitis [26]. In this model, the epithelial cell barrier is impaired chemically by DSS administration, leading to the disruption of intestinal homeostasis and dysbiosis of gut microbiota. As shown in Fig. 1a, mice were fed a standard chow diet (CD) or an isocaloric diet where BL was supplemented at a ratio of 2.5% (Additional file 2: Table S2). There was no significant difference in the food intake or water intake between control and BL-fed mice (Additional file 1: Fig. S1A and B), suggesting that BL had no adverse effects on the eating and drinking habits. During the colitis induction, supplementation of BL significantly protected against DSS-induced body weight loss, reduced disease activity index (DAI) scores, and colon shortening (Fig. 1b–d). Supplementation of BL also significantly protected against DSS-induced intestinal permeability (Fig. 1e). Since inflammatory cytokines are crucial factors during the onset of colitis [27], we further assessed the impact of BL on inflammatory cytokines. BL feeding significantly increased the levels of interleukin (IL)-4 and IL-10 and decreased the level of TNF-α in serum of DSS-treated mice (Additional file 1: Fig. S1C-E). A similar change was also observed in colonic tissues (Additional file 1: Fig. S1F-H). These data indicate that BL effectively alleviates colitis symptoms in DSS-treated mice.

Ulcerative colitis is typically associated with dysbiosis of gut microbiota [14]. To study whether BL could prevent against DSS-induced gut microbiota dysbiosis, we analyzed the gut microbiota composition by performing a 16S rRNA gene amplicon sequencing in the colonic contents. The overall structure of gut microbiota that was investigated by UniFrac-based principal coordinates analysis (PCoA) showed that the CD+DSS group displayed a shift clustering of bacterial composition, which was distinct from either CD or BL group (PERMANOVA, *P* < 0.001) (Fig. 1f), suggesting that the dysbiosis of gut microbiota was induced by DSS treatment. Notably, BL protected against DSS-induced dysbiosis as evidenced by that the microbiota in BL+DSS group was more closely clustered to CD and BL group relative to CD+DSS group (PERMANOVA, *P* < 0.001) (Fig. 1f). Consistently, the α-diversity analysis with Chao and Shannon index revealed that BL significantly prevented DSS-induced decrease in bacterial richness and diversity (Fig. 1g). We then further analyzed the gut bacterial composition at phylum and family levels. BL significantly reduced DSS-induced increase in the relative abundance of *Proteobacteria* (Wilcoxon rank-sum test, *P* < 0.01) (Fig. 1h). Meanwhile, further analysis at the family level revealed that DSS treatment caused a dramatic increase in the abundance of *Enterobacteriaceae*, a common signature of the gut microbiota dysbiosis [28], which was also significantly suppressed by BL supplementation (from 20% ± 4% to 1.5% ± 0.4%, Wilcoxon rank-sum test, *P* < 0.0001) (Fig. 1i).

To evaluate whether a preventive or a therapeutic setting contributes to the protection against DSS-induced colitis, we performed another experiment in which mice were supplemented with BL before (beBL) or during (duBL) the DSS treatment (Additional file 1: Fig. S1I). Notably, mice with beBL treatment exhibited improved body weight loss, DAI severity, and colon length shortening compared with mice with duBL treatment, producing effects similar to those in BL group (Additional file 1: Fig. S1J-L). Similarly, histological analysis of colonic sections revealed that mice in the CD+DSS and duBL+DSS groups exhibited serious injuries including crypt distortion, goblet cell loss, and inflammatory cell infiltration in lamina propria and submucosa, which were markedly abrogated in the beBL+DSS and BL+DSS groups (Additional file 1: Fig. S1M and N). Taken together, these results suggest that dietary supplementation of BL protects against DSS-induced colitis and gut microbiota dysbiosis, and these effects may largely depend on a preventive manner.

### BL enhances colonic motility and improves mucosal barrier function

**Fig. 2:**
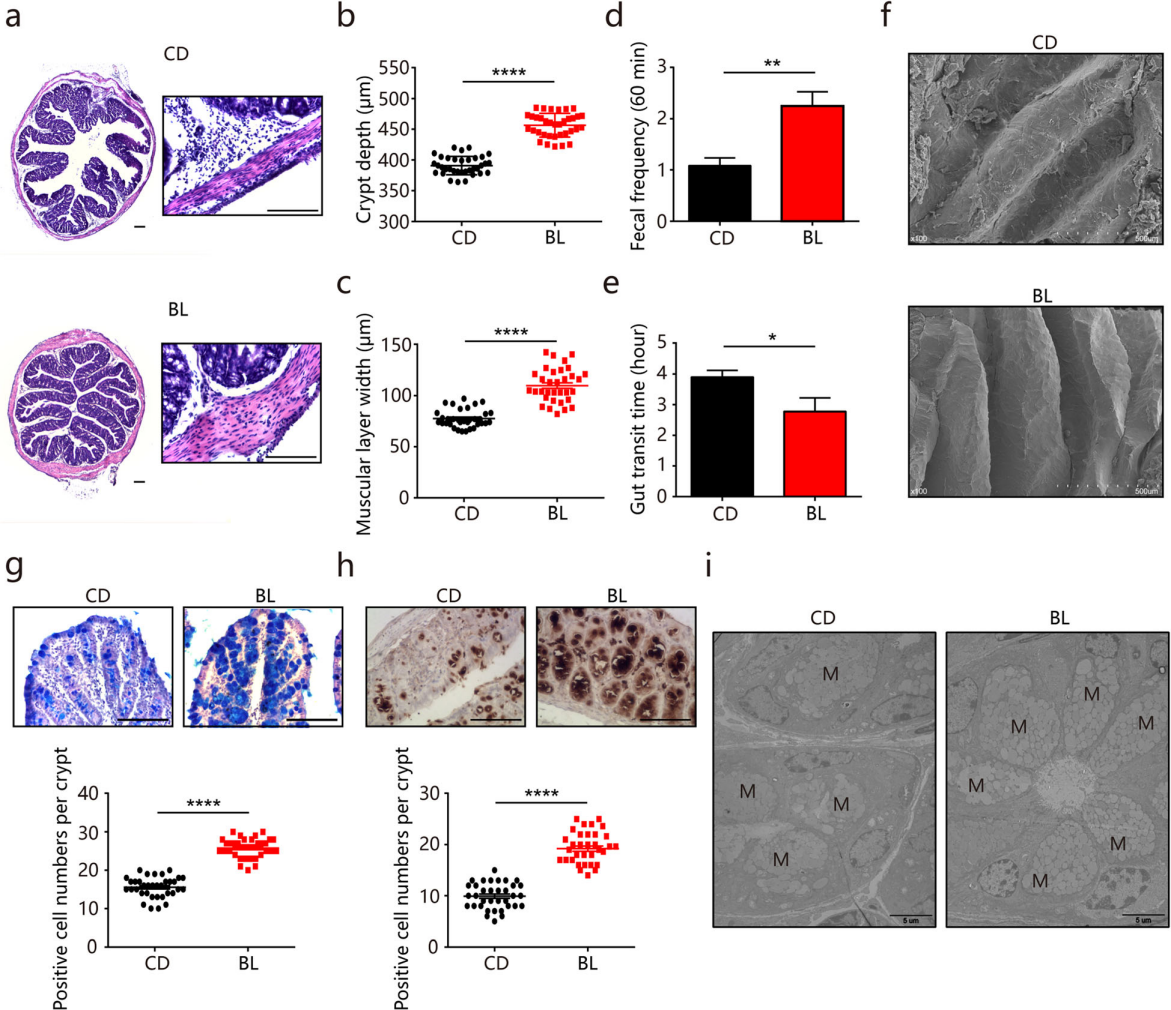
Barley leaf (BL) enhances colonic motility and improves mucosal barrier function. **a**–**i** Mice were fed with a standard chow diet (CD) or an isocaloric BL-supplemented diet for two weeks. **a** Representative images of hematoxylin and eosin-stained colonic sections. The right panels were enlarged from the left panels. Scale bar = 200 μm (left) and 100 μm (right). **b** Crypt height and **c** muscular layer width in the colon of CD- and BL-fed mice were quantified (*n* = 12). **d** Fecal pellet output and **e** gut transit time of CD- and BL-fed mice were measured (*n* = 8). **f** Representative images of scanning electron microscopy of the colonic mucosal surface. Scale bar = 500 μm. **g** Representative images of alcian blue-stained colonic sections and the number of mucus­producing goblet cells was quantified (*n* = 12). Scale bar = 100 μm. **h** Representative images of Muc2-stained colon sections and the number of Muc-2-positive goblet cells was quantified (*n* = 12). Scale bar = 100 μm. **i** Representative images of transmission electron microscopy of the mucin granules (M) in colonic goblet cells. Scale bar = 5 μm. Data are pooled from three independent experiments. Data are mean ± SEM. *P < 0.05, **P < 0.01, and ****P < 0.0001. Statistical analysis was performed using Student’s *t* test

We speculated that the preventive impacts facilitated by BL on DSS-induced colitis might be explained by the possibility that dietary supplementation of BL could improve the intestinal functions before colitis induction. Investigation of intestinal morphology showed that BL-fed mice had a significant increase in crypt height and muscular layer width in the colon compared with control mice (Fig. 2a–c). Nevertheless, the altered morphology was not observed in the small intestine **(**Additional file 1: Fig. S2A**)**. The coordinated contractions of the muscular layer facilitate the motility of the intestine [29]. The increased colonic muscular layer width in BL-fed mice prompted us to speculate whether BL feeding could have an influence on intestinal motility. Compared with control mice, increased stool frequency and shortened gut transit time were observed in BL-fed mice (Fig. 2 d and e). Moreover, scanning electron microscope images revealed that BL-fed mice displayed drastic alterations of ultra-morphology as indicated by obvious edges and corners on the colonic mucosal surface (Fig. 2f), suggesting that BL might facilitate protective effects on colitis prevention through improving intestinal barrier functions. However, there were no significant differences in the expression of genes encoding for tight junction proteins such as zonula occludens1 (*ZO1*), *Occludin*, *Claudin2*, and *Claudin4* and antimicrobial peptides such as regenerating islet-derived 3b (*Reg3b*), *Reg3g*, *Defensin4*, and matrix metalloprotease 7 (*Mmp7*) (Additional file 1: Fig. S2B-I) between BL-fed and control mice. In addition, both groups of mice displayed comparable levels of macrophages and neutrophil infiltration, as evidenced by no major differences in the expression levels of F4/80 and Ly6G in colonic tissues (Additional file 1: Fig. S2J and K).

We next focused on the colonic mucus layer to better understand the impacts of BL on mucosal barrier functions. As shown in Fig. 2g, alcian blue staining showed that the number of mucus­producing goblet cells was significantly increased in the colon of BL-fed mice compared with that of control mice. Of note, mucin granules in the goblet cells of BL-fed mice appeared to present enhanced apical exocytosis and secretion on the mucosal surface, which were not observed in control mice (Fig. 2g). Mucin2 (Muc2), the most abundant mucin protein that forms the mucus layer, is heavily glycosylated and secreted by goblet cells [10]. Immunohistochemical analysis revealed that there were also more Muc-2-positive goblet cells in the colon of BL-fed mice compared with that of control mice (Fig. 2h). Closer examination by transmission electron microscopy showed that the theca of colonic goblet cells in BL-fed mice contained large mucin granules (Fig. 2i), suggesting the enhanced biogenesis and accumulation of mucin protein in colonic goblet cells. These results suggest that BL improves intestinal functions possibly by promoting colonic motility and enhancing mucosal barriers.

### BL alters gut gene expression profile and activates the PPARγ signaling pathway

**Fig. 3:**
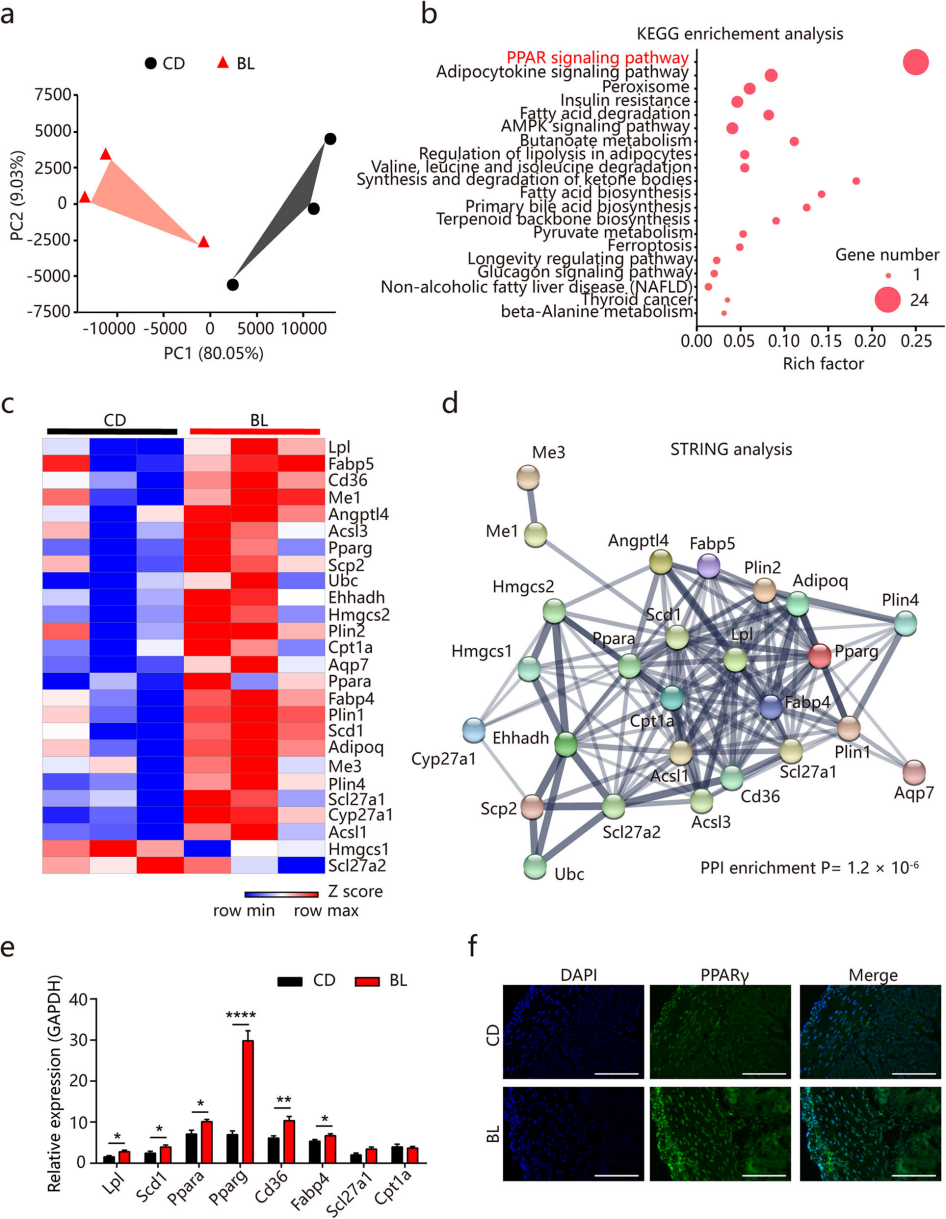
Barley leaf (BL) alters gene expression in mouse colon. **a**–**f** Mice were fed a standard chow diet (CD) or an isocaloric BL-supplemented diet for 2 weeks (*n* = 3). **a** Principle component analysis (PCA) of transcriptional profiling of the mouse colonic tissues. Permutational multivariate analysis of variance (PERMANOVA): *F* = 6.00, Df = 2, *P* = 0.017. **b** Kyoto Encyclopedia of Genes and Genomes (KEGG) pathway enrichment analysis of the most significantly changed pathways. **c** Heat map of differentially expressed genes in peroxisome proliferators-activated receptor (PPAR) signaling pathway. Genes with fold changes of > 1 and false discovery rates of < 0.05 were considered to be differentially expressed. **d** Search tool for recurring instances of neighboring genes (STRING) network visualization of the genes in differentially expressed genes in the PPAR signaling pathway. Edges represent protein-protein associations. **e** Real-time PCR assay for the differentially expressed genes in PPAR signaling pathway (*n* = 10). **f** Immunofluorescent analysis of PPARγ (green) in mouse colonic sections. Nuclei were stained with DAPI (blue). Scale bar = 100 μm. Data are pooled from three independent experiments (**e**). Data are representative of two independent experiments (**f**). Data are mean ± SEM. *P < 0.05, **P < 0.01, and ****P < 0.0001. Statistical analysis was performed using Student’s *t* test

To further delineate the mechanisms underlying the improvements of intestinal motility and mucosal barrier functions in BL-fed mice, we performed genome-wide transcriptional profiling of the whole colonic tissues for RNA-sequencing. There were significant differences in the transcriptomes between BL-fed and control mice (Fig. 3a). Based on significant false discovery rate *P* value criteria (fold change > 1 and *P* < 0.05), 1735 significantly altered genes were identified. Among them, 912 genes were upregulated and 823 genes were downregulated in the colon of BL-fed mice versus control mice. Kyoto Encyclopedia of Genes and Genomes (KEGG) pathway analysis of differentially expressed genes showed enrichment in a series of signaling pathways (Fig. 3b). Of note, PPAR signaling was the most significantly enriched functional pathway in BL-fed mice (Fig. 3b). Interestingly, 26 significantly changed genes that were enriched in PPAR signaling were mainly involved in nutrient metabolisms, such as lipid biosynthesis (*Scd1*), lipid degradation (*Lpl*, *Adipoq*), lipid storage (*Plin1*, *Plin2*, *Plin4*), lipid transport (*Scp2*, *Slc27a1*, *Slc27a2*), lipid binding (*Fabp4*, *Fabp5*, *CD36*, *Ppara*, *Pparg*), β-oxidation (*Cpt1a*, *Acsl1*, *Acsl3*, *Ehhadh*), and cholesterol metabolism (*Hmgcs1*, *Hmgcs2*, *Cyp27a1*) (Fig. 3c). This was consistent with a previous study demonstrating that lipid metabolism is closely associated with improvements of mucosal barrier functions [30]. Furthermore, search tool for recurring instances of neighboring genes (STRING) analysis was used to depict interaction networks of the significantly changed genes (Fig. 3d). Using quantitative real-time PCR, we confirmed the mRNA expression of key genes in the PPAR signaling pathway and found that *Pparg* was the most significantly changed gene in BL-fed mice compared with control mice (Fig. 3e). Consequently, the increased protein expression of PPARγ in colonic tissues of BL-fed mice was further confirmed by immunofluorescence imaging (Fig. 3f). Overall, these results suggest that BL feeding results in alteration of genes associated with nutrient metabolism and the activation of PPARγ signaling may contribute to the impacts of BL on intestinal functions.

**Fig. 4:**
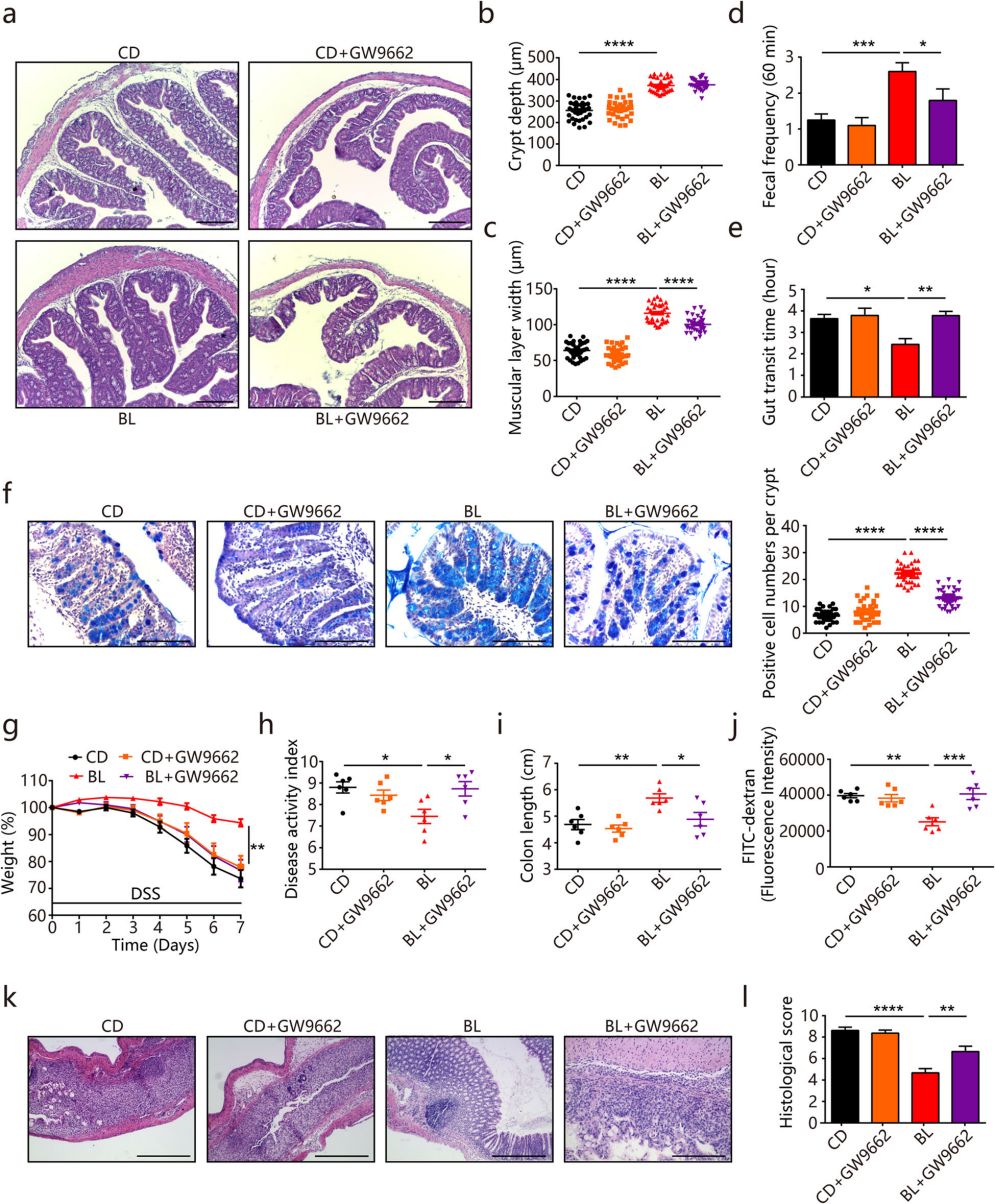
Barley leaf (BL) improves intestinal functions through the PPARγ signaling activation. **a**–**l** Mice were fed a chow diet (CD) or an isocaloric BL-supplemented diet for 2 weeks. PPARγ antagonist GW9662 was administered with 3 mg/kg/day intragastrically. Colitis was induced by administering 2.5% DSS dissolved in drinking water for 7 days. **a** Representative images of hematoxylin and eosin-stained colonic sections. Scale bar = 100 μm. **b** Crypt height and **c** muscular layer width in the colon of mice from different mouse groups were quantified (*n* = 8). **d** Fecal pellet output and **e** gut transit time from different mouse groups were measured (*n* = 8). **f** Representative images of alcian blue-stained colonic sections and the number of mucus­producing goblet cells was quantified (*n* = 8). Scale bar = 200 μm. **g** Percentage body weight change, **h** diseases activity scores, **i** colon lengths, and **j** intestinal permeability from different mouse groups were measured (*n* = 6). **k** Representative images of hematoxylin and eosin-stained colonic sections and **l** histological scores. Scale bar = 200 μm. Data are pooled from three independent experiments. Data are mean ± SEM. *P < 0.05, **P < 0.01, ***P < 0.001 and ****P < 0.0001. For body weight change, a repeated measure two-way analysis of variance (ANOVA) was performed and the rest of the statistics was performed with one-way ANOVA followed by Tukey’s multiple comparisons test

PPARγ signaling pathway plays an essential role in the regulation of intestinal homeostasis [19]. To assess whether the PPARγ signaling pathway was associated with improved intestinal functions, we treated mice with PPARγ signaling antagonist GW9662 by daily oral gavage [19]. Consistent with the above impacts on intestinal morphology, BL feeding resulted in a higher crypt height and muscular layer width in the colon **(**Fig. 4a–c). Moreover, increased stool frequency and shortened gut transit time were also observed in BL-fed mice compared with control mice (Fig. 4d and e). However, when the PPARγ signaling was suppressed by GW9662, the effects of BL on muscular layer width, stool frequency, intestinal transit time were abolished (Fig. 4c–e). PPARγ signaling inhibition also abrogated the beneficial effects of BL on mucosal barrier functions because BL failed to increase the number of mucus­producing goblet cells in GW9662-treated mice (Fig. 4f).

To determine whether PPARγ signaling activation was required for the impacts of BL on colitis protection, we examined colitis symptoms in the DSS-induced colitis model. As expected, BL-fed mice were protected from DSS-induced body weight loss, increased DAI scores, and intestinal permeability, as well as colon shortening (Fig. 4g–j). However, PPARγ signaling inhibition by GW9662 abolished the protective effects of BL on DSS-induced colitis (Fig. 4g–j). Additionally, the protective effects of BL on DSS-induced epithelial damage were also abrogated by GW9662 treatment (Fig. 4k and l). Collectively, these findings indicate that BL protects against DSS-induced colitis possibly through activating the PPARγ signaling pathway.

### BL promotes the enrichment of gut microbiota-derived purine metabolites

**Fig. 5:**
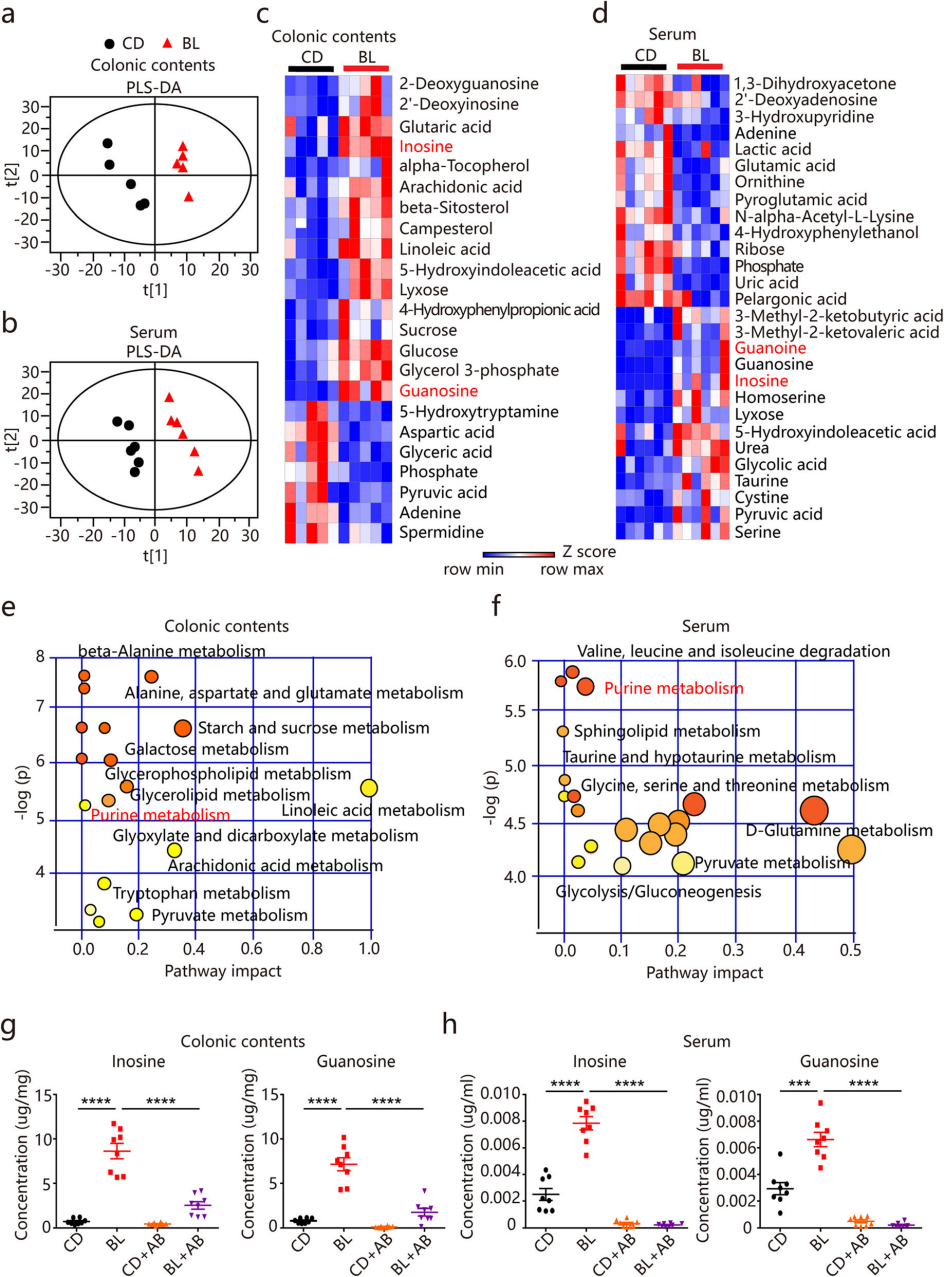
Barley leaf (BL) alters metabolic profiling and enriched microbiota-derived purine metabolites. **a**–**h** Mice were fed with a standard chow diet (CD) or an isocaloric BL-supplemented diet for two weeks. **a**, **b** Partial least-squares discriminant analysis (PLS-DA) of metabolomic profiles in colonic contents (*n* = 5) and serum (*n* = 6) of CD- and BL-fed mice. **c**, **d** Heat map of significantly altered metabolites in colonic contents (*n* = 5) and serum (*n* = 6) of CD- and BL-fed mice. Metabolites with the Variable importance in the projection (VIP) value of PLS-DA model >1 and the P values of Student’s *t* test < 0.05 were considered to be significantly different. **e**, **f** Pathway enrichment analysis of the significantly altered metabolites in colonic contents and serum of CD- and BL-fed mice. The *x*-axis represents the pathway impact, and the *y*-axis represents the pathway enrichment. **g**, **h** Concentrations of inosine and guanosine in colonic contents and serum of CD- and BL-fed mice (*n* = 8). AB, antibiotics. Data are pooled from three independent experiments (**g** and **h**). Data are mean ± SEM. ***P < 0.001 and ****P < 0.0001. Statistical analysis was performed by one-way ANOVA followed by Tukey’s multiple comparisons test

Dietary nutrients have a great impact on the regulation of commensal microbial metabolites, which are considered pivotal mediators of host-microbiota interactions [16]. We hypothesized that the potential mechanisms by which BL influenced the intestinal functions are via the secondary metabolites generated during microbial fermentation in the gut. To assess metabolic alterations in response to BL feeding, untargeted metabolomic profiles were generated on colonic contents and serum from BL-fed and control mice by high-resolution gas chromatography and mass spectrometry (GC-MS). As shown in Fig. 5 a and b, the partial least squares discrimination analysis (PLS-DA) model exhibited a significant separation of clusters between the two mouse groups. Heat map analysis showed that BL feeding resulted in dramatic alteration of metabolites, with a total of 23 metabolites (16 upregulated and 7 downregulated) and 28 metabolites (14 upregulated and 14 downregulated) were significantly altered in mouse colonic contents and serum, respectively (Fig. 5c and d). The metabolomic map revealed the significantly enriched pathway affected by BL feeding and most of these pathways were associated with energy and nutrient metabolisms such as glucose, lipid, and amino acid metabolism (Fig. 5e and f). Notably, purine metabolism was the pathway that was significantly influenced by BL in both the colonic contents and serum (Fig. 5e and f). Specifically, we found that inosine and guanosine, two major intermediate metabolites involved in purine metabolism, were the most significantly increased metabolites in response to BL feeding in both colonic contents and serum (Fig. 5c and d).

It has been known that inosine and guanosine are important foodstuff additives that are obtained at the industrial level mainly by microbial fermentation [31]. We further speculated that the gut microbiota might be associated with the enrichment of inosine and guanosine in BL-fed mice. To test this hypothesis, we treated BL-fed and control mice with broad-spectrum antibiotics to eliminate the gut microbiota and further examined the levels of inosine and guanosine by targeted analysis. Compared with the control mice, the BL-fed mice displayed significantly increased levels of inosine and guanosine in both colonic contents and serum (Fig. 5g and h). However, antibiotic treatment strongly decreased the levels of these two purine metabolites (Fig. 5g and h).

Based on the role of gut microbiota in modulating the enrichment of inosine and guanosine in BL-fed mice, we hypothesized that the gut microbiota might also play an important role in regulating the impacts of BL on mice intestinal functions. Accordingly, we performed untargeted metabolomics analysis to study differentially expressed small molecule metabolites in colonic tissues between BL-fed and control mice. As expected, the principal components analysis (PCA) model and the PLS-DA model exhibited a distinct clustering of metabolites between the two groups, indicating that BL supplementation resulted in metabolic alterations in the colon **(**Additional file 1: Fig. S3A**)**. Heat map analysis showed that 45 metabolites were significantly altered by BL feeding **(**Additional file 1: Fig. S3B). Among them, 14 metabolites were increased and 31 metabolites were decreased in BL-fed mice versus control mice **(**Additional file 1: Fig. S3B). Interestingly, the majority of metabolites that contribute highly to the metabolic distinction were relevant to energy pathways. Specifically, a significant decrease of glycolysis substrates such as glucose, glucose-6-phosphate, and fructose-6-phosphate, as well as a significant increase of glycolysis product lactic acid, was identified in BL-fed mice compared with control mice **(**Additional file 1: Fig. S3C-F). This was consistent with the above results of increased intestinal motility in BL-fed mice (Fig. 2), suggesting that BL promoted colonic motility possibly through the enhanced glycolysis pathway. Notably, when the gut microbiota was suppressed by antibiotic treatment, the metabolic alterations caused by BL were abolished **(**Additional file 1: Fig. S3C-F). Thus, these data suggest that BL induces metabolic reprogramming in colonic tissues via a gut microbiota-dependent manner.

To directly assess the role of the gut microbiota in BL-mediated enrichment of purine metabolites, we measured the concentrations of inosine and guanosine after the incubation of BL with fresh mice feces for in vitro fermentation assay under strict anaerobic conditions **(**Additional file 1: Fig. S4A**)**. Fermentation of BL by the gut microbiota resulted in significantly increased levels of inosine and guanosine, which were not changed in vehicle batch cultures **(**Additional file 1: Fig. S4B and C**)**.

To elucidate the mechanism responsible for the accumulation of inosine and guanosine, we performed 16S rRNA gene sequencing to analyze the bacterial composition in batch cultures at 12h and 24h. BL fermentation resulted in marked alterations of the bacterial communities, as revealed by a distinct clustering of microbiota composition for 12h and 24h BL fermentation group compared with their respective vehicle control group (PERMANOVA, *P* < 0.05) **(**Additional file 1: Fig. S4D). Decreased α-diversity was observed at each time point in BL fermentation group, suggesting losses of particular bacterial taxa induced by BL fermentation **(**Additional file 1: Fig. S4E and F**)**. Analyses of the microbiota at the phylum level revealed a significant increase in the relative abundance of *Firmicutes* and a decrease in the relative abundance of *Bacteroidetes* in the BL fermentation group than in the vehicle control group **(**Additional file 1: Fig. S4G and H**)**. Notably, BL increased the relative abundance of *Lactobacillus* from 0.41% ± 0.02% to 25.89% ± 7.46% and from 0.42% ± 0.15% to 37.92% ± 2.92% by 12 h and 24 h of fermentation, respectively **(**Additional file 1: Fig. S4I and J). Finally, Pearson’s correlation analyses showed that the abundance of *Lactobacillus* had positive relationships with the concentrations of inosine (Pearson’s correlation coefficients, *R* = 0.8883, *P* < 0.0001) and guanosine (Pearson’s correlation coefficients, *R* = 0.9266, *P* < 0.01) respectively in anaerobic cultures **(**Additional file 1: Fig. S4K and L**)**. These data indicate that the enriched *Lactobacillus* may contribute to the enrichment of inosine and guanosine, which was consistent with the results of a previous study [32].

### Inosine activates PPARγ signaling in human colon epithelial cells

**Fig. 6:**
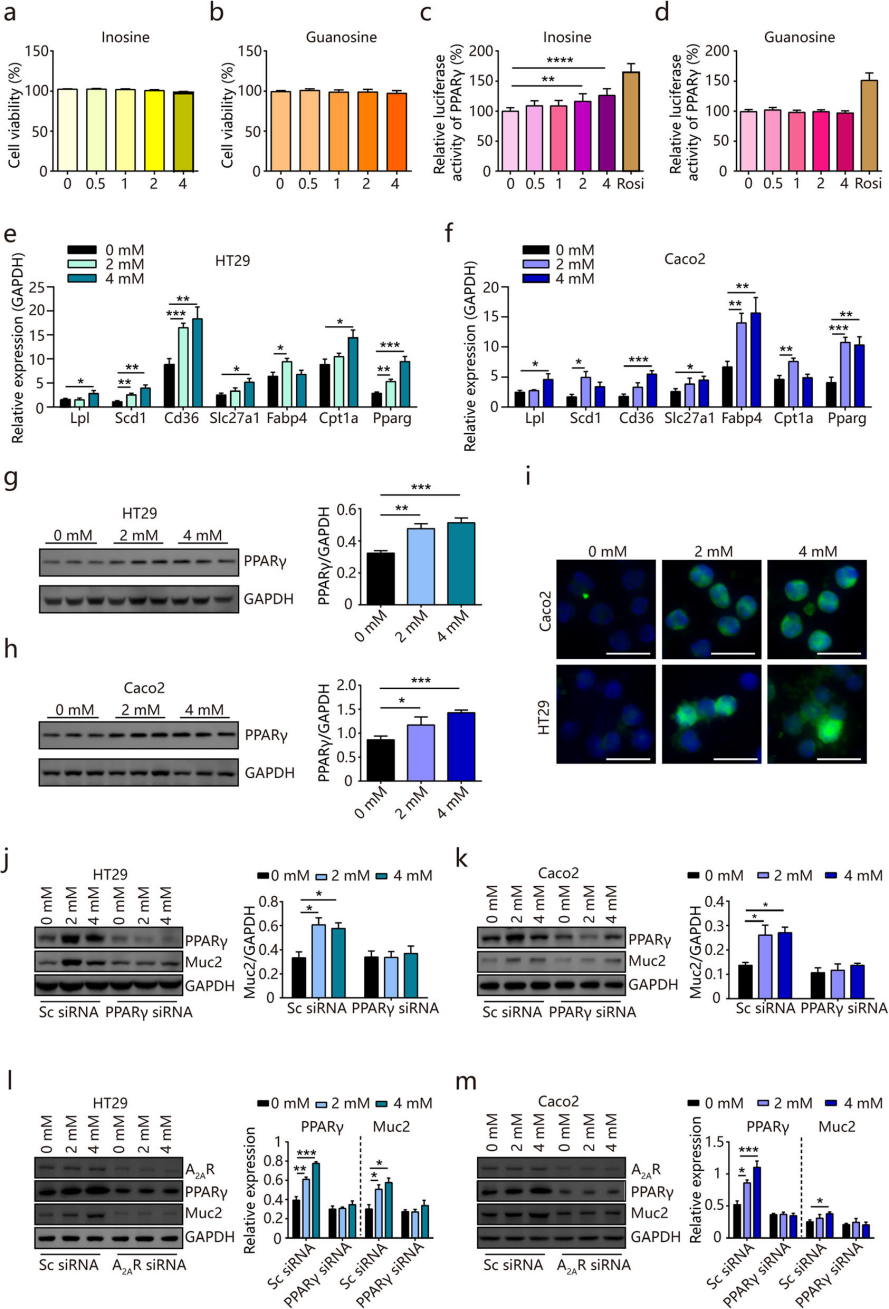
Inosine, but not guanosine, activates PPARγ signaling pathway in human colon epithelial cells. **a**, **b** HT29 cells were treated with inosine (0.5, 1, 2, and 4 mM) or guanosine (0.5, 1, 2, and 4 mM) for 24 h and cell viability analysis was measured by CCK-8 assay. **c**, **d** HT29 cells were treated with inosine (0.5, 1, 2, and 4 mM) or guanosine (0.5, 1, 2, and 4 mM) or 10 μM rosiglitazone (Rosi) for 24 h and PPARγ activity was measured by luciferase reporter gene assay. **e**, **f** HT29 and Caco2 cells were treated with inosine (2 and 4 mM) for 24h and the expression of genes in the PPARγ signaling pathway was examined by real-time PCR assay. **g**, **h** HT29 and Caco2 cells were treated with inosine (2 and 4 mM) for 24h and the protein level of PPARγ was examined by western blot. **i** HT29 and Caco2 cells were treated with inosine (2 and 4 mM) for 24h and the protein level of PPARγ (green) was examined by immunofluorescence analysis. Nuclei were stained with DAPI (blue). Scale bar = 20 μm. **j**, **k** HT29 and Caco2 cells were treated with inosine (2 and 4 mM) for 24 h and PPARγ was knocked down using siRNA. The protein levels of PPARγ and Muc2 were examined by western blot. **l**, **m** HT29 and Caco2 cells were treated with inosine (2 and 4 mM) for 24h and A_2A_R was knocked down using siRNA. The protein levels of A_2A_R, PPARγ, and Muc2 were examined by western blot. Scrambled (Sc) siRNA transfections were used as controls. Immunoblots were quantified using ImageJ software. Data are pooled from three independent experiments (**a**–**f**). Data are representative of two independent experiments (**g**-**m**). Data are mean ± SEM. *P < 0.05, **P < 0.01, ***P < 0.001, and ****P < 0.0001. Statistical analysis was performed using Student’s *t* test

Given that microbial metabolites are in close contact with gut epithelium, we hypothesized that the metabolites could have a direct impact on intestinal epithelial cell functions. To examine such effects, we assessed the functional activity of inosine and guanosine in vitro. As shown in Fig. 6a and b, both metabolites with different concentrations (0.5, 1, 2, and 4 mM) showed no discernible toxicity in human colon epithelial HT-29 cells (Fig. 6a and b). Next, a luciferase-based system was used to detect the bioactivity of inosine and guanosine on PPARγ signaling. Interestingly, the PPARγ-luciferase activity was significantly increased in cells treated with inosine or in cells treated with rosiglitazone, but not in guanosine-treated cells (Fig. 6c and d), suggesting that inosine but not guanosine might be the metabolite responsible for BL-induced PPARγ signaling activation. We also observed significantly increased expression of key genes in the PPARγ signaling pathway, such as lipid degradation gene *Lpl*, lipid biosynthesis gene *Scd1*, lipid binding genes *CD36*, *Fabp4*, lipid transport gene *Slc27a1*, and β-oxidation gene *Cpt1a* after inosine treatment in both HT29 cells and another colon epithelial cell line, Caco2 (Fig. 6e and f). The elevated protein level of PPARγ was further confirmed by western blot and immunofluorescence imaging (Fig. 6g–i). To investigate whether PPARγ was required for inosine-mediated induction of mucin protein, PPARγ expression was suppressed using siRNA and the expression of Muc2 was examined. Interestingly, the level of Muc2 was significantly increased by inosine treatment. However, inosine failed to induce Muc2 in PPARγ knockdown cells (Fig. 6j and k). Because inosine has been reported as a ligand for adenosine 2A receptor (A_2A_R) [21], we tested whether inosine mediated PPARγ signaling activation through A_2A_R. As shown in Fig. 6l and m, inosine failed to induce PPARγ and Muc2 in A_2A_R knockdown cells. These results suggest that inosine induces the expression of mucin protein at least in part through the A_2A_R/PPARγ pathway.

### Inosine improves intestinal functions and protects against colitis via A_2A_R/PPARγ

**Fig. 7:**
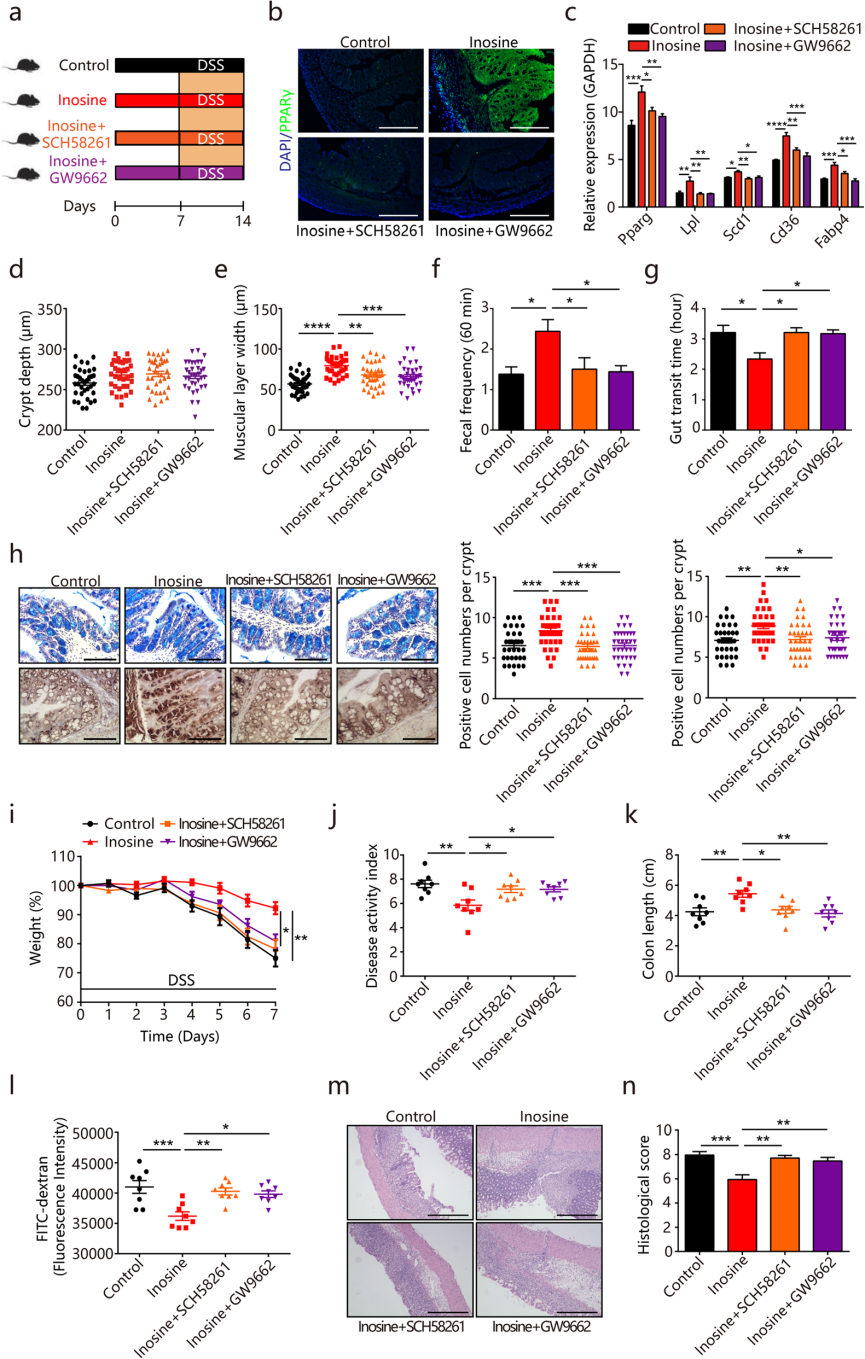
Inosine protects against dextran sulfate sodium (DSS)-induced colitis through the A_2A_R/PPARγ signaling pathway. **a**–**n** Mice were treated intragastrically with 800 mg/kg/day of inosine dissolved in PBS at the concentration of 40 mg/ml. PPARγ antagonist GW9662 were administered at 3 mg/kg/day intragastrically and A_2A_R antagonist SCH58261 were treated with 2 mg/kg/day intraperitoneally. Colitis was induced by administering 2.5% DSS dissolved in drinking water for 7 days. **a** Study design of in vivo mouse experiment. **b** Immunofluorescent analysis of PPARγ (green) in mouse colonic sections from different mouse groups. Nuclei were stained with DAPI (blue). Scale bar = 100 μm. **c** Real-time PCR assay for the differentially expressed genes in PPAR signaling pathway (*n* = 8). **d** Crypt height and **e** muscular layer width in the colon of mice from different mouse groups were quantified (*n* = 12). **f** Fecal pellet output and **g** gut transit time from different mouse groups were measured (*n* = 8). **h** Representative alcian blue-stained and Muc2-stained colon sections from different mouse groups. The number of mucus­producing goblet cells and Muc-2-positive goblet cells was quantified (*n* = 12). Scale bar = 100 μm. **i** Percentage body weight change, **j** diseases activity scores, **k** colon lengths, and **l** intestinal permeability were measured from different mouse groups (*n* = 8). **m** Representative images of hematoxylin and eosin-stained colonic sections and **n** histological scores. Scale bar = 100 μm. Data are pooled from three independent experiments. Data are mean ± SEM. *P < 0.05, **P < 0.01, ***P < 0.001 and ****P < 0.0001. For body weight change, a repeated measure two-way analysis of variance (ANOVA) was performed and the rest of the statistics was performed with one-way ANOVA followed by Tukey’s multiple comparisons test

We next determined the in vivo role of A_2A_R**/**PPARγ as a mediator of the beneficial effects induced by inosine (Fig. 7a). Consistent with the above in vitro data, inosine treatment resulted in significantly increased expression of PPARγ and its target genes (*Lpl*, *Scd1*, *CD36*, and *Fabp4*) in colonic tissues (Fig. 7b and c). Meanwhile, treatment of inosine partially phenocopied the effects of BL on intestinal morphology, as evidenced by significantly increased muscular layer width in the colon of inosine-treated mice (Fig. 7d and e). Inosine also enhanced gut motility via increasing the stool frequency and decreasing the gut transit time (Fig. 7f and g). In addition, our results revealed that inosine treatment improved mucosal barrier functions by increasing the number of mucus-producing goblet cells (Fig. 7h). However, inosine failed to induce these effects in mice treated with GW9662 or A_2A_R-specific antagonist SCH58261 (Fig. 7e–h). We then examined whether inosine contributed to the protective effects of BL on DSS-induced colitis. As shown in Fig. 7i–k, inosine reduced DSS-induced body weight loss, attenuated DAI scores, and colon shortening. We also observed that inosine effectively enhanced barrier function as evidenced by improved intestinal permeability and histological damage in DSS-treated mice (Fig. 7l–n). Nevertheless, the beneficial effects of inosine on colitis were reversed when mice were treated with GW9662 or SCH58261 (Fig. 7i–n). Out data thus reveal that inosine exerts the protective effects against colitis partly through A_2A_R**/**PPARγ signaling.

## Discussion

Ulcerative colitis is emerging as a health-threatening disease in the world [4]. Conventional drug-based therapies have led to substantial economic costs and certain side effects [33]. Hence, there remains an urgent need for the development of effective and safe treatment agents. BL, an herbal-based dietary supplement in Traditional Chinese Medicine, is an attractive candidate due to its potent health benefits on intestinal functions. In this study, we found that dietary supplementation of BL protected against DSS-induced colitis and gut microbiota dysbiosis. Moreover, we identified for the first time that inosine, a gut microbial purine metabolite, contributed to the protective effects of BL against DSS-induced colitis by improving A_2A_R**/**PPARγ-dependent mucosal barrier functions **(**Additional file 1: Fig. S5**)**. Thus, our results suggest that the microbiota-inosine-A_2A_R**/**PPARγ axis could have tremendous potential for preventing and treating intestinal inflammatory disorders, such as colitis.

The intestinal tract is colonized by a large and diverse microbial community, which plays a critical role in the maintenance of intestinal homeostasis [34–36]. A growing body of evidences has suggested that gut microbiota dysbiosis appears to be associated with the pathogenesis of ulcerative colitis [37]. For instance, compared with healthy subjects, the gut microbiota of inflammatory bowel disease patients exhibited a reduction in microbial diversity [38, 39]. In line with these findings, our 16S rRNA gene sequencing data also showed that DSS treatment caused a reduced gut microbial richness and diversity in mice, which was effectively rescued by BL supplementation. The dysbiosis-associated reduction in gut microbial diversity may be due to a shift in the balance between commensal and potentially pathogenic microorganisms. Indeed, it has been reported that a healthy gut microbiota composition is dominated by anaerobic *Firmicutes* and *Bacteroidetes*, while microbiota dysbiosis is featured by the expansion of facultative anaerobic pathogens belonging to the *Enterobacteriaceae* family [28, 40]. In the present study, we found that BL significantly suppressed the increased abundance of *Enterobacteriaceae* in DSS-treated mice. The mechanism by which BL inhibited DSS-induced gut microbiota dysbiosis may partially result from its ability to activate PPARγ signaling in the colon, because a recent study reports that PPARγ activation can limit gut lumen oxygen level by driving β-oxidation in colonic epithelial cells, thereby inhibiting the expansion of pathogenic *Enterobacteriaceae* [19].

Apart from the gut microbiota, other crucial factors that are also involved in the maintenance of intestinal homeostasis include intestinal motility and barriers, because dysfunction of intestinal motility and mucosal barriers are common symptoms frequently presented in patients with inflammatory bowel disease [41, 42]. In this study, we found that the protection of BL against DSS-induced colitis was through a preventive manner which might largely attribute to its beneficial effects on intestinal functions. The increased crypt height and muscular layer width observed in BL-treated mice indicated that dietary supplementation of BL induced enhanced intestinal motility before colitis induction, which was also confirmed by the increased stool frequency and shortened gut transit time. It was noted that the BL-induced morphological alterations were specific to the large intestine where a high density of microorganisms is present, suggesting the role of gut microbiota in the regulation of these effects. Additionally, metabolomics analysis showed that BL caused metabolic reprogramming in mice colon characterized by the promotion of the glycolysis process, which may partially explain the enhanced intestinal motility. However, antibiotic treatment abrogated the metabolic alterations in BL-fed mice, suggesting that BL might promote intestinal motility in a gut microbiota-dependent manner.

We next applied RNA-sequencing to investigate the underlying mechanisms of BL on intestinal functions. Notably, we noted a significant increase in the expression of genes involved in biosynthesis, degradation, storage, transport, and binding of lipid. A previous study has revealed an essential role of de novo lipogenesis enzyme fatty acid synthase in maintaining intestinal barrier function through modulating the palmitoylation of Muc2 [30]. Thus, the increased expression of genes associated with lipid metabolism may explain the improvements of mucosal barrier function in BL-fed mice. Further functional analysis showed that PPAR signaling was the most significantly enriched functional pathway in response to BL feeding. PPARγ is an essential nuclear receptor that is mainly expressed in adipose tissue and is involved in the regulation of insulin resistance by controlling the expression of a large number of regulatory genes in lipid metabolism. Notably, PPARγ is also highly expressed in the colon, while its expression is impaired in patients with ulcerative colitis [43]. The mechanisms by which PPARγ signaling modulate the intestinal homeostasis have been reported to be associated with the modulation of colon epithelium cell differentiation [44], adaptive immune effector T cell function [45], and innate immune antimicrobial response [46]. Moreover, PPARγ signaling also plays an important role in regulating the impacts of high-fat diet on the mucosal immune defenses in the small intestine [47]. In line with these findings, our data showed that inhibition of PPARγ signaling by GW9662 abrogated the protective effects of BL on colonic motility and mucosal barrier, as well as the protection against DSS-induced colitis.

Given the pivotal role in the modulation of intestinal homeostasis, PPARγ has been recognized as an important target for the treatment of inflammatory bowel disease. For instance, troglitazone and rosiglitazone are the two PPARγ synthetic ligands that can dramatically reduce disease severity in experimental colitis models and in patients with ulcerative colitis [48–50]. Moreover, PPARγ is also an important target of 5-aminosalicylic acid, one of the oldest anti-inflammatory agents used for the treatment of inflammatory bowel disease [51]. Interestingly, many natural ligands that present in food such as conjugated linoleic acid have been reported to efficiently prevent the development of colitis through PPARγ signaling activation [52]. Notably, conjugated linoleic acid is a metabolite that can be generated by gut bacteria *Lactobacillus*, indicating the involvement of gut microbiota in the regulation of PPARγ signaling [53]. Correspondingly, another study has demonstrated that the microbial fermentation product butyrate induces PPARγ signaling activation in vitro and in vivo [54]. To search for the potential agonists responsible for BL-induced PPARγ signaling activation, an untargeted metabolomics analysis was performed. We found that supplementation of BL resulted in the enrichment of purine nucleotides, inosine, and guanosine in both colonic contents and serum. We examined their activities by using in vitro functional analysis. Our results revealed that the activity and expression of PPARγ could be induced by the treatment of inosine but not guanosine in human colon epithelial cells, suggesting that inosine may be a potent agonist for PPARγ signaling activation. Furthermore, exogenous treatment with inosine activated colonic PPARγ signaling and protected against DSS-induced colitis through improving mucosal barrier functions. Notably, no obvious toxicity and side effects were observed in our in vitro and in vivo experiments, suggesting that inosine has the potential to be developed as a promising agent for the treatment of colitis. Nevertheless, further study will be required to determine the efficacy and safety of inosine in ulcerative colitis patients.

It has been known that inosine and guanosine are important foodstuff additives that have synergistic effects with monosodium glutamate to increase the intensity of the “umami” taste [55]. At the industrial level, microbial fermentation technology is the most efficient method for their production. By using genetic engineering, several bacterial strains such as *Bacillus subtilis* [56], *Corynebacterium ammoniagenes* [57], and *Escherichia coli* [58] have been applied to increase the production of these purine nucleotides. It should be noted that the production of inosine and guanosine can also be obtained by metabolic engineering of a filamentous fungus *Ashbya gossypii* [31], suggesting the potential use of both eukaryotic and prokaryotic microorganisms in industrial nucleoside production. In the present study, antibiotic treatment and anaerobic fermentation experiments confirm our hypothesis that gut microbiota plays an indispensable role in promoting the enrichment of purine metabolites, which was consistent with the results of the recent study [22]. Interestingly, the 16S rRNA gene sequencing of bacterial composition revealed an increased abundance of *Lactobacillus* during BL fermentation, suggesting the role of *Lactobacillus* in regulating the enrichment of these purine metabolites. This is supported by a recent study demonstrating that *Lactobacillus reuteri* can inhibit regulatory T cell deficiency-induced autoimmunity via inosine-A_2A_R signaling [32]. In the present study, the inhibition of A_2A_R by the antagonist ligand SCH58261 blocked the effects of inosine on PPARγ signaling activation and mucosal barrier improvements, suggesting that A_2A_R may be a novel target for the treatment of intestinal disorders [59].

## Conclusions

In the present study, we demonstrate that dietary supplementation of BL can protect against DSS-induced colitis and microbiota dysbiosis by promoting intestinal motility and improving mucosal barrier functions. In addition, BL promotes the enrichment of microbiota-derived purine metabolite inosine, which induces A_2A_R/PPARγ signaling activation and contributes to the impacts of BL on the improvements of intestinal functions. Our results highlight that BL and inosine might be used as efficacious supplements in individuals with intestinal dysfunction.

## Methods

### Preparation of BL powder

BL powder used in this study was crude powder without any extraction process and was supplied by Hebei Biotechnology Co., Ltd. (Jiaxing, China). Briefly, fresh leaves of *Hordeum vulgare* L. (cultivated in Hangzhou, China) were washed with water, cut into pieces, and dried in a freeze dryer OE-950 (Labor, MIM, Budapest, Hungary) at − 60 °C for 24 h. The dried BL was pulverized with a blender (KA-2610, Jworld Tech, Korea) for 1 min, screened through a 300-mesh sieve, and stored at − 20 °C until use. The proximate analysis of the BL powder was performed by Pony Testing International Group (Beijing, China) **(**Additional file 2: Table S1**)**.

### Animal experiments

Eight-week-old female C57Bl/6J mice were obtained from Vital River Laboratory Animal Technology (Beijing, China) and maintained under specific-pathogen-free (SPF) facility with a 12-h light and dark cycle. All mice were adapted to the laboratory conditions with ad libitum access to food and water for at least one week. Animal experiments were conducted in accordance with the Guidelines for Animal Experimentation of Peking University Health Science Center (Beijing, China), and the protocols were approved by the Animal Ethics Committee of this institution. Mice were randomly distributed and fed with either a standard chow diet (CD) or an isocaloric diet where BL was supplemented at a ratio of 2.5%, a dosage translated from a previous human intervention study [24]. The composition of two diets was listed in Additional file 2: Table S2 and prepared by Hfk Biotech Co, Ltd (Beijing, China).

For the treatment with PPARγ antagonist GW9662 or A_2A_R antagonist SCH58261, mice were treated with 3 mg/kg/day of GW9662 intragastrically [19] or treated with 2 mg/kg/day of SCH58261 intraperitoneally [32].

For antibiotic experiment, a combination of neomycin (100 mg/l), streptomycin (50 mg/l), penicillin (100 mg/l), vancomycin (50 mg/l), and metronidazole (100 mg/l) were administered in the drinking water.

For the treatment with inosine, mice were treated intragastrically with 800 mg/kg/day of inosine (Sigma-Aldrich) dissolved in PBS at the concentration of 40 mg/ml [32].

### Dextran Sodium Sulfate (DSS)-induced colitis

Colitis was induced by administering 2.5% DSS (molecular weight 36,000–50,000 kDa; MP Biomedicals) dissolved in drinking water for 7 days. The mice were weighed daily and monitored for signs of stool consistency and rectal bleeding. Evaluation of disease activity index was performed by combining the parameters of weight loss, stool consistency, and rectal bleeding as described previously [60]. The disease activity index was the mean of the total score of the three parameters. Mice were sacrificed on day 7 and colon length was measured.

### Assessment of intestinal permeability

Briefly, fluorescein isothiocyanate (FITC)-dextran (4 kDa; Sigma) was dissolved in PBS at a concentration of 100 mg/ml. After mice were fasted for 4 h and orally administrated with FITC-dextran (60 mg/100 gm body weight). Blood was collected following another 4 h and was centrifuged at 1000 rpm for 20 min. Serum was collected and fluorescence was quantified at an excitation wavelength of 485 nm and 535 nm emission wavelength.

### 16S rRNA gene sequencing and analysis

Total DNA was extracted from colonic contents or fecal culture samples using a QIAamp-DNA Stool Mini Kit (Qiagen, Hilden, Germany). The integrity of the extracted DNA was examined by electrophoresis in 1% (wt/vol) agarose gels. Based on the quantity and the quality of the DNA extracted, samples were selected to perform the consequent sequencing.

DNA samples were used as the template for PCR amplification of the V3–V4 region of bacterial 16S rRNA genes. PCR amplification was performed on ABI GeneAmp®9700 PCR System (AppliedBiosystems, Foster City, CA, USA) and the PCR amplification products were quantified with a QuantiFluor^TM^-ST Handheld Fluorometer with UV/Blue Channels (Promega Corporation, Madison, WI, USA).

Sequencing of the PCR amplification products was performed on an Illumina Miseq platform at Majorbio Bio-Pharm Technology Co., Ltd. (Shanghai, China). Briefly, the 16S rRNA gene sequencing data was filtered and trimmed and further classified into operational taxonomic units (OTUs) within a 0.03 difference (equivalent to 97% similarity). A representative set of sequences was then generated and was assigned taxonomy using the SILVA database (Release115 http://www.arb-silva.de). Analyses for rarefaction curves and calculation of richness estimators and diversity indices were performed using the MOTHUR program. Taxonomic community structure and phylogeny were analyzed through visualization of the data sets of the microbial diversities and abundances of different samples.

### Inflammatory cytokine analysis

Cytokine levels in serum and colonic tissues were detected by enzyme-linked immunosorbent assay (ELISA) kit (Nanjing Jiancheng Bioengineering Institute Co., Ltd. Nanjing, China) according to the manufacturer's instructions.

### Histological evaluation

Collected colon samples were fixed in 4% paraformaldehyde and embedded in paraffin. Paraffin-embedded colonic tissues were sectioned (4μm in thicknesses) and were subjected to hematoxylin and eosin (HE) and alcian blue (AB) staining by using commercial kits (Beijing Solarbio Technology Co., Ltd. Beijing, China) according to the manufacturer's instructions. The crypt height and muscular layer width in the large intestine were measured using ImageJ (Media Cybernetics, Inc., Rockville, MD, USA). Mucus­producing goblet cells were observed and counted under light microscope (Leica DM500). Six microscopic fields of every section of the testes were randomly selected.

Histological pathology was scored according to a previously established scoring system, as follows: crypt damage (0–4 scale), severity of inflammation (0–3 scale), and depth of injury (0–3 scale) [61].

### Intestinal motility assessment

The intestinal motility was determined through evaluating the intestinal transit time and defecation frequency. After mice were fasted for 4 h, individual mouse was gavaged with the 10 μL/g of 6% carmine solution (in 0.5% methylcellulose). The time from gavage to initial appearance of carmine in the feces was recorded as the gut transit time for a given mouse.

Defecation frequency was recorded by separating mice into a sterile cage and the cumulative fecal pellets within 60 min were recorded as the corresponding defecation number for a given mouse.

### Scanning electron microscopy

Colonic tissues were fixed with 2.5% PBS-diluted glutaraldehyde. After being rinsed three times in PBS, samples were post-fixed in 1% osmium tetroxide for 1 h, dehydrated in alcohol, and then critical-point-dried using CO_2_. The samples were coated with gold and observed in a Hitachi S-3400 scanning electron microscope (Hitachi, Japan).

### Transmission electron microscopy

Colonic tissues were fixed with 2.5% glutaraldehyde. PBS was used to remove excess fixative. Samples were then fixed with 1% osmium tetroxide at 4 °C for 2 h, dehydrated in acetone. Samples were finally infiltrated with 1:1 propylene oxide and EPON resin for 1 h followed by overnight infiltration in 100% EPON's resin. The tissues were embedded in flat molds in 100% EPON for 36 h at 60 °C. Ultra-thin sections of 70 nm were stained with uranyl acetate and lead citrate (10 min each) and viewed under an H-7650 transmission electron microscope (Hitachi, Japan).

### Immunohistochemistry and immunofluorescence

Following deparaffinization and rehydration, colonic sections were blocked with 5% bovine serum albumin for 30 min at room temperature and then washed with PBS. Tissue sections were incubated with primary antibody Muc-2 (Santa Cruz, sc-515032), Ly6G (Abcam, ab25377), and F4/80 (Abcam, ab6640) overnight at 4 °C. Slides were washed three times in PBS before applying peroxidase-conjugated secondary antibody (Invitrogen) for 2 h at room temperature.

For immunofluorescence staining, paraffin-embedded colonic sections or cells were incubated with primary antibody PPARγ (Abcam, ab59256) at 4 °C overnight. The next day, Alexa Fluor488 donkey antirabbit secondary antibody (Invitrogen) was used.

### Quantitative real-time PCR

Total RNA of colonic tissues was isolated by using TRIZOL reagent (Invitrogen, USA). The quantity and purity of RNA were assessed by absorbance at 260 nm and 280 nm. Reverse transcription of 10 μl RNA was performed using PrimeScript RT Master Kit (Takara, Japan). Gene expression was measured in a MyiQ single color Real-Time PCR detection system (Bio-Rad) with SYBR Real-time PCR Kit (Takara, Japan). GAPDH was used as endogenous control. Average Ct values from triplicate analyses were normalized from average Ct values of GAPDH. The primer sequences are described and synthesized by Sunbiotech Co. (Beijing, China) **(**Additional file 2: Table S3**)**.

### RNA sequencing

Colon tissues were collected and the total RNA was extracted by using TRIZOL reagent (Invitrogen, USA). RNA quality was evaluated by electrophoresis using an Agilent 2100 Bioanalyzer (Agilent Technologies, San Diego, CA, USA). Samples with RNA integrity numbers (RINs) > 9.4 and with 260/280 nm absorbance ratios from 1.9 to 2.1 were used for the construction of RNA-Seq libraries. Libraries were constructed using the TruSeq™ RNA Sample Prep kit (Illumina, San Diego, CA, USA) according to the manufacturer’s instructions.

Sequencing of the libraries was performed on an Illumina HiSeq2000 instrument by Shanghai Majorbio Biopharm Biotechnology (Shanghai, China), and individually assessed for quality using FastQC. Analysis of differential expression was carried out using DESeq2 [62]. Statistical significance was assessed using a negative binomial Wald test, then corrected for multiple hypothesis testing with the Benjamini-Hochberg method. Functional enrichment cluster analysis was performed for Kyoto Encyclopedia of Genes and Genomes (KEGG) pathway enrichment analysis.

### Non-targeted metabolomics

Gas chromatography-mass spectrometry (GC-MS)-based metabolomics was performed by ProfLeader Biotech (Shanghai, China). Briefly, serum samples were separated from the blood by centrifugation and stored at −80°C. Colonic contents or colon tissue samples mixed with water were vortexed prior to centrifugation. The supernatant was transferred to a GC vial containing internal standards. The mixture was dried under a gentle nitrogen stream and then added with methoxyamine hydrochloride in pyridine. The resultant mixture was vortexed vigorously and incubated at 37°C for 90 min. Derivatization was performed by adding BSTFA (with 1% TMCS) into the mixture. The derivatized samples were analyzed by an Agilent 7890A gas chromatography system coupled to an Agilent 5975C inert MSD system (Agilent Technologies, CA, USA). A HP-5MS fused-silica capillary column was utilized to separate the derivatives. Helium was used as a carrier gas at a constant flow rate through the column. The samples were analyzed in a random sequence.

The acquired GC/MS data were imported to SIMCA Statistical Analysis (version 13.0, Umetrics AB, Umeå, Sweden), where multivariate statistical analysis including principal component analysis (PCA) and partial least-squares discriminant analysis (PLS-DA) were performed. The differential metabolites were determined by the combination of the variable importance in the projection (VIP) value (> 1) of the PLS-DA model and the *P* values (<0.05) from two-tailed Student’s *t* test on the normalized peak intensities. Fold change was calculated as a binary logarithm of the average normalized peak area ratio between the two groups. The structural identification of differential metabolites was performed using AMDIS software, where the purified mass spectra were automatically matched with an in-house standard library including retention time and mass spectra, Agilent Fiehn GC/MS Metabolomics RTL library, and Golm Metabolome Database, respectively.

### Quantitative analysis of purine metabolites

Serum, colonic contents, or fecal culture samples were mixed with distilled water containing internal standard (4-aminosalicylic acid). The mixture was extracted by ethyl acetate. Following centrifugation, the top layer was transferred and evaporated to dryness under a nitrogen stream. The dry residue was reconstituted in acetonitrile and BSTFA (with 1% TMCS) and derivatized to perform GC-MS analysis. A mixed standard solution containing inosine and guanosine stock standard solutions was prepared at a concentration of 100 μg/mL for quantitation.

### In vitro anaerobic culturing

Fresh mice feces (0.5 g) from 8-week-old male C57Bl/6 mice were resuspended in 10 ml sterile PBS and co-cultured with BL powder with a final concentration of 10 mg/ml under anaerobic conditions (10% H_2_, 10% CO_2_, 80% N_2_). After incubation for 12 and 24 h, cultured samples were collected and used for metabolites detection and bacterial composition analysis.

### Cell culture and treatment

Human colon epithelial carcinoma cell lines HT29 and Caco2 were purchased from the American Type Culture Collection (ATCC). Cells were maintained in Dulbecco’s modified Eagle’s medium (DMEM) supplemented with 10% fetal bovine serum (FBS) and 1% penicillin/streptomycin in a humidified incubator (5% CO_2_, 95% air, 37 °C). Cells were grown until 80 to 90% confluence and treated for the indicated time periods with inosine (Sigma-Aldrich) and guanosine (Sigma-Aldrich) from a stock in PBS to a final concentration of 0.5, 1, 2, and 4mM. Cell viability was evaluated with the Cell Counting Kit (CCK)-8 cell viability assay kit from GenMed Inc. (Shanghai, China) according to the manufacture’s instruction.

### Luciferase reporter assay

PPARγ-luciferase plasmid vector was purchased from RiboBio Co., Ltd (Guangzhou, China). HT29 cells were transiently transfected using a lipofectamine 3000 reagent (ThermoFisher Scientific). Briefly, 1 × 10^5^ cells were seeded on 6-well plates and grown for 24 h. The transfection complex containing 1 μg of plasmid DNA and transfection reagent was added to each well in absence of FBS. After 6 h, a medium containing 10% FBS was added and cells were treated with inosine and guanosine (0.5, 1, 2, and 4 mM) for 24 h. The luciferase activity was measured with Luciferase Assay System (Promega) using a luminometer (Perkin Elmer, Covina, CA).

### Western blot analysis

Cells were lysed with lysis buffer with a 1% protease inhibitor cocktail to harvest total cellular protein. The concentration of extracted protein was quantified by bicinchoninic acid (BCA) protein assay kit (Beyotime). An equal amount of protein sample was loaded on sodium dodecyl sulfate-polyacrylamide gel electrophoresis gel and then transferred to a polyvinylidene difluoride membrane (Millipore, Billerica, MA, USA). After the membrane was blocked with skimmed milk, it was incubated with primary antibodies against Muc2 (Santa Cruz, sc-515032), PPARγ (Abcam, ab59256), A_2A_R (Abcam, ab3461), and GAPDH (Abcam, ab8245). The next day, secondary antibodies conjugated with Horseradish peroxidase were probed for 2–3 h. Protein signals were detected with enhanced ECL chemiluminescence reagent based on the manufacturer’s instructions.

### Small interfering RNA

The siRNA against PPARγ or A_2A_R was purchased from GenePharma (Shanghai, China). For knockdown experiments, 1 × 10^5^ HT29 and Caco2 cells were plated in 6-well plates and grown for 24 h. The PPARγ, A_2A_R, or control-siRNA was transfected into cells using a lipofectamine 3000 reagent (ThermoFisher Scientific). After 24 h of transfections, cells were induced with inosine (0, 2, and 4 mM) for 24 h. And then cells were lysed using RIPA buffer and the protein expression of A_2A_R, PPARγ, or Muc2 was detected by western blot.

### Statistical analysis

Statistical analysis was performed using Prism 6.0 (GraphPad Software, CA). Data are presented as means ± SEM. Significant differences between the two groups were evaluated by a two-tailed unpaired Student’s *t* test. Significant differences in more than two groups were evaluated by one-way or two-way analysis of variance (ANOVA) followed by Tukey’s multiple comparisons test.

When analyzing gut microbiota sequencing data, a two-tailed Wilcoxon rank-sum test by R Project was performed. When analyzing the differences of abundance distributions among metabolites, a Mann-Whitney *U* test with Benjamini-Hochberg false discovery rate correction was performed. The differences were considered to be significant at *P* < 0.05.

## Supplementary Information

**Additional file 1: Figure S1.** Barley leaf (BL) protects against dextran sulphate sodium (DSS)-induced colitis through a preventive manner. **Figure S2.** Barley leaf (BL) does not affect histological structures in the small intestine. **Figure S3.** The gut microbiota is required for barley leaf (BL)-induced metabolic reprograming in colonic tissues. **Figure S4.** Barley leaf (BL) fermentation alters the gut microbiota composition and mediates enrichment of purine metabolites. **Figure S5.** Proposed model for the preventive effects of barley leaf (BL) against dextran sulfate sodium (DSS)-induced colitis.

**Additional file 2: Table S1.** The macronutrient composition of the BL powder in the present study. **Table S2.** The composition of CD and BL diet. **Table S3.** List of primers used in this study.

## Data Availability

The sequences generated in this study are stored in the National Center for Biotechnology Information (NCBI) and the project numbers are PRJNA511491 and PRJNA511549. Additionally, all other data is contained within the main manuscript and supplemental files.
