# Supplementary material for: Gut microbiota-derived inosine from dietary barley leaf supplementation attenuates colitis through PPARγ signaling activation
Source: Microbiome. 2021 Apr 5;9:83. doi: 10.1186/s40168-021-01028-7 (PMC8022418; doi:10.1186/s40168-021-01028-7)
Supplement: Supplementary file 2 — Additional file 1: Figure S1. Barley leaf (BL) protects against dextran sulphate sodium (DSS)-induced colitis through a preventive manner. Figure S2. Barley leaf (BL) does not affect histological structures in the small intestine. Figure S3. The gut microbiota is required for barley leaf (BL)-induced metabolic reprograming in colonic tissues. Figure S4. Barley leaf (BL) fermentation alters the gut microbiota composition and mediates enrichment of purine metabolites. Figure S5. Proposed model for the preventive effects of barley leaf (BL) against dextran sulfate sodium (DSS)-induced colitis. [file 40168_2021_1028_MOESM2_ESM.docx]

**Additional file 1**

**Gut microbiota-derived inosine from dietary barley leaf supplementation attenuates colitis through PPARγ signaling activation**

Daotong Li, Yu Feng, Meiling Tian, Junfu Ji, Xiaosong Hu, Fang Chen^[[1]](#footnote-1)^*

**This file includes:**

Fig. S1. Barley leaf (BL) protects against dextran sulphate sodium (DSS)-induced colitis through a preventive manner.

Fig. S2. Barley leaf (BL) does not affect histological structures in the small intestine.

Fig. S3. The gut microbiota is required for barley leaf (BL)-induced metabolic reprograming in colonic tissues.

Fig. S4. Barley leaf (BL) fermentation alters the gut microbiota composition and mediates enrichment of purine metabolites.

Fig. S5 Proposed model for the preventive effects of barley leaf (BL) against dextran sulfate sodium (DSS)-induced colitis.

**Fig. S1**

**
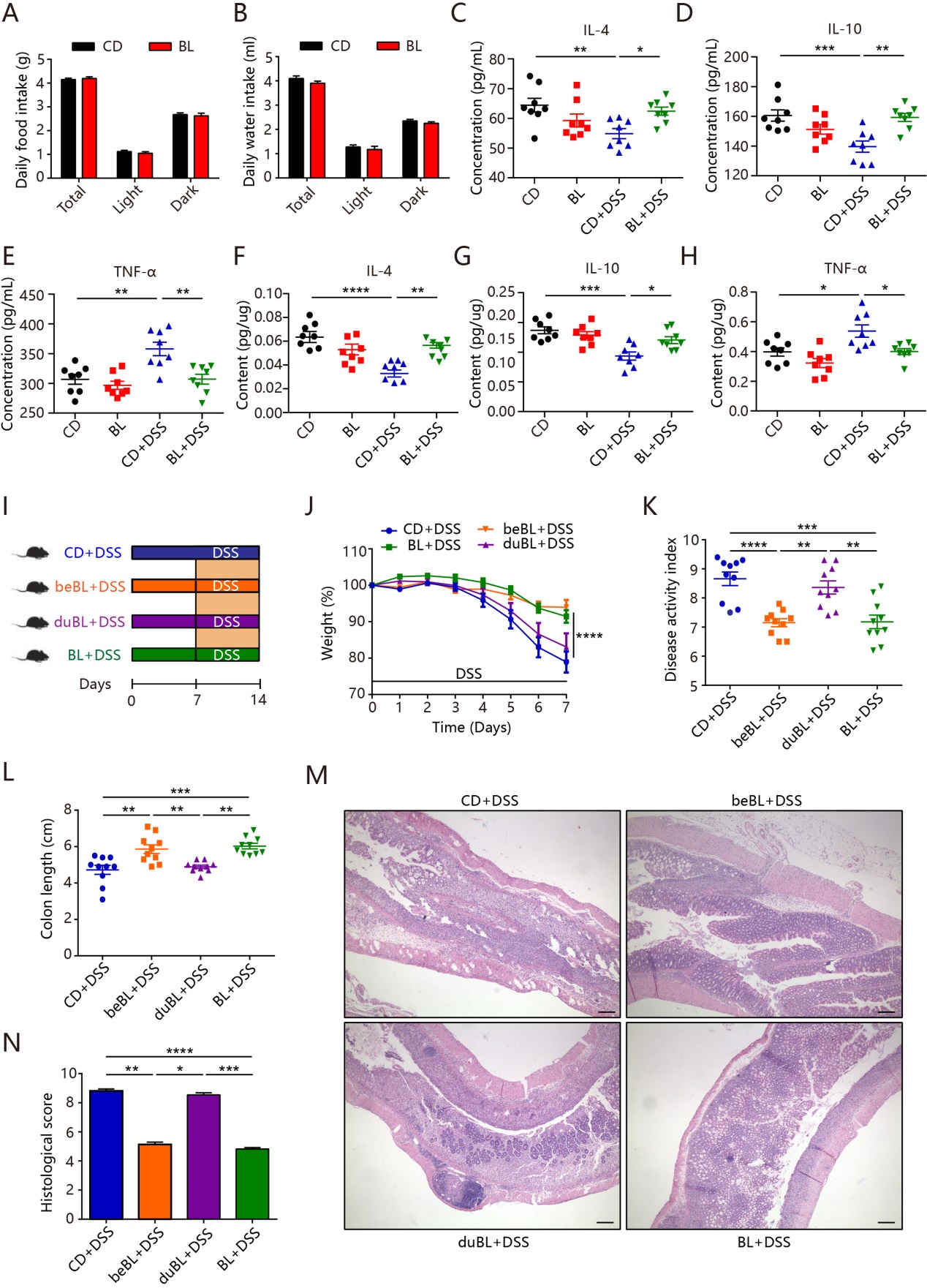
**

**Fig. S1 Barley leaf (BL) protects against dextran sulphate sodium (DSS)-induced colitis through a preventive manner.** (A-H) Mice were fed with a standard chow diet (CD) or an isocaloric BL-supplemented diet for two weeks. Colitis was induced by administering 2.5% DSS dissolved in drinking water for 7 days. (A) Daily food intake and (B) daily water intake were measured in CD- and BL-fed mice. (C-E) The levels of interleukin (IL)-4, IL-10 and tumour necrosis factor-α (TNF-α) in the serum of CD- and BL-fed mice with or without DSS treatment (n = 8). (F-H) The levels of IL-4, IL-10 and TNF-α in the colonic tissues of CD- and BL-fed mice with or without DSS treatment (n = 8). (I-N) Mice were supplemented with BL before (beBL) or during (duBL) the DSS induction. Colitis was induced by administering 2.5% DSS dissolved in drinking water for 7 days. (I) Study design of in vivo mouse experiment. (J) Percentage body weight change, (K) diseases activity scores and (L) colon lengths were measured from different mouse groups (n = 10). (M) Representative images of hematoxylin and eosin-stained colonic sections and (N) histological scores. Data are pooled from three independent experiments. Scale bar = 200 μm. Data are mean ± SEM. *P < 0.05, **P < 0.01, ***P < 0.001 and ****P < 0.0001. For body weight change, a repeated measure two-way analysis of variance (ANOVA) was performed and the rest of the statistics was performed with one-way ANOVA followed by Tukey’s multiple comparison’s test.

**Fig. S2**

**
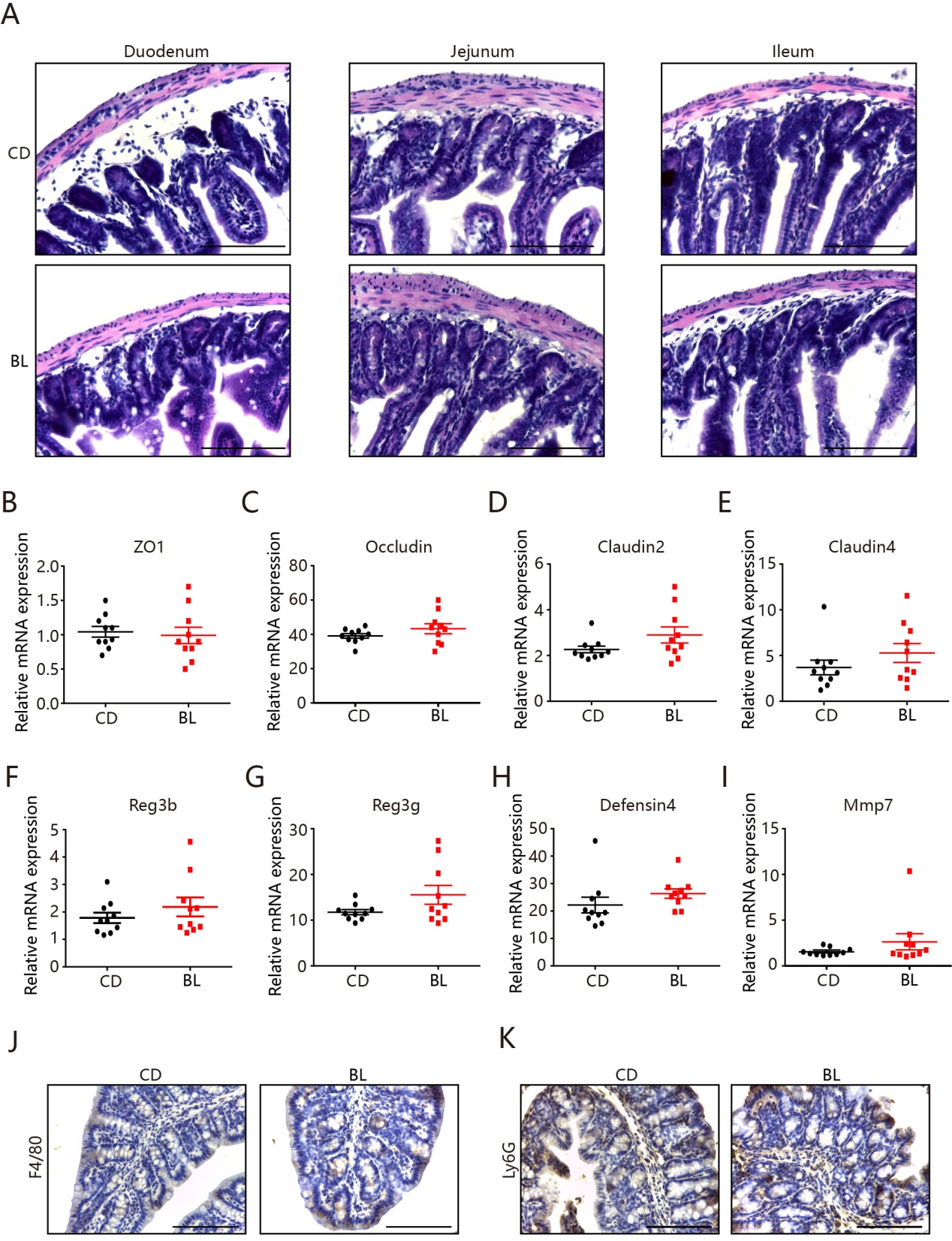
**

**Fig. S2 Barley leaf (BL) does not affect histological structures in the small intestine.** Mice were fed a chow diet (CD) or an isocaloric BL-supplemented diet for two weeks. (A) Representative images of hematoxylin and eosin-stained colon. Scale bar = 100 μm. (B-E) Real-time PCR assay for the expression of genes encoding for tight junction proteins (n = 10). (F-I) Real-time PCR assay for the expression of genes encoding for antimicrobial peptides (n = 10). (J and K) Immunohistochemistry analysis of F4/80 for macrophages and Ly6G for neutrophils in mouse colonic sections of CD- and BL-fed mice. Scale bar = 100 μm. Data are pooled from three independent experiments (B-I). Data are representative of two independent experiments (A, J and K). Data are mean ± SEM. Statistical analysis was performed using Student’s *t* test. ZO1, zonula occludens 1; Reg3b, regenerating islet derived 3b; Mmp7, matrix metalloprotease 7.

**Fig. S3**

**
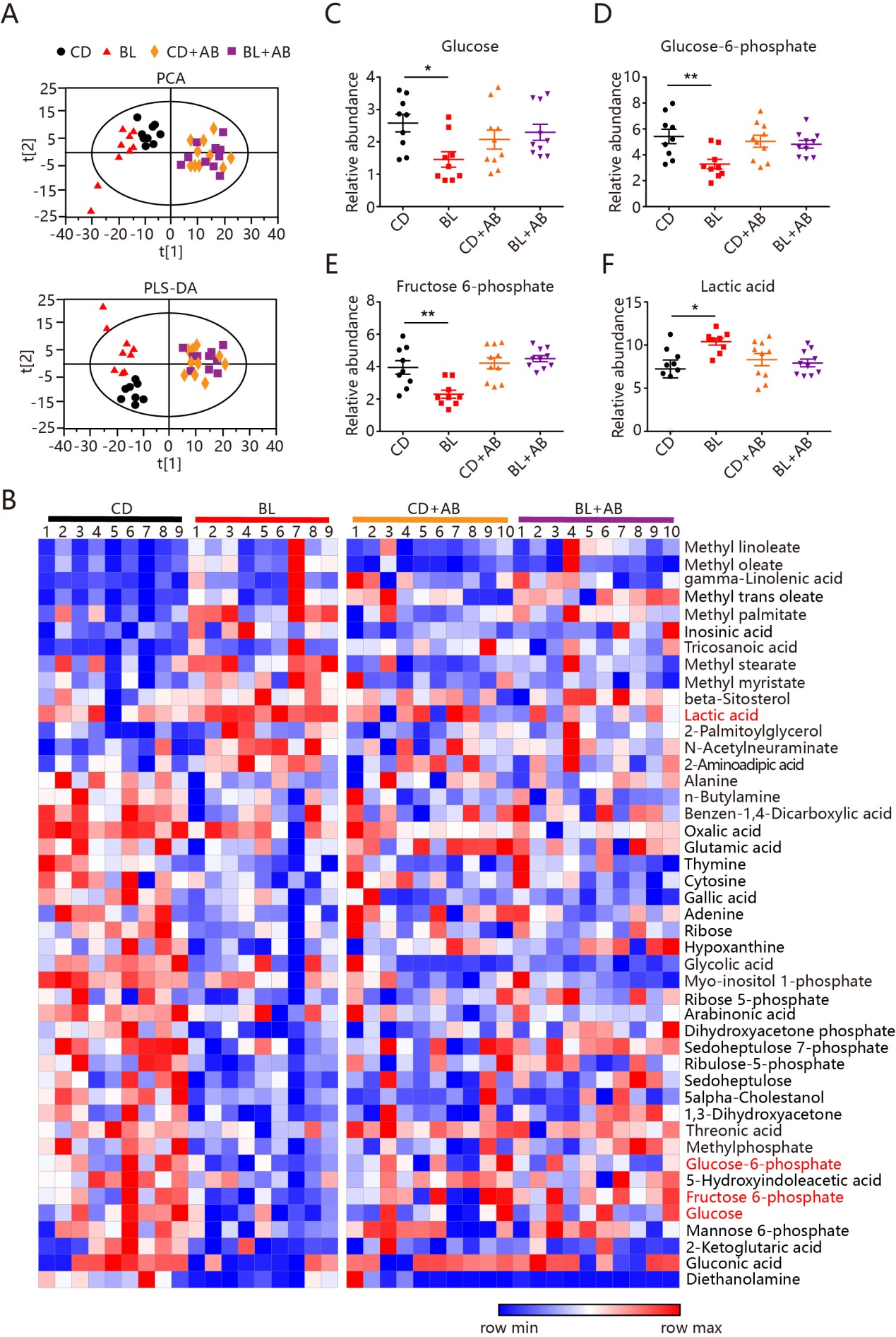
**

**Fig. S3 The gut microbiota is required for barley leaf (BL)-induced metabolic reprograming in colonic tissues.** Mice were fed a chow diet (CD) or an isocaloric BL-supplemented diet for two weeks. A combination of antibiotics (AB) including neomycin (100 mg/l), streptomycin (50 mg/l), penicillin (100 mg/l), vancomycin (50 mg/l) and metronidazole (100 mg/l) were administered in the drinking water. (A) Principle component analysis (PCA) (upper) and partial least-squares discriminant analysis (PLS-DA) (bottom) of metabolomic profiles in mouse colonic tissues of different mouse groups (n = 9-10). (B) Heat map of significantly altered metabolites in mouse colonic tissues of different mouse groups (n = 9-10). (C-F) The relative abundance of glucose, glucose-6-phosphate, fructose-6-phosphate and lactic acid in mouse colonic tissues of different mouse groups (n = 9-10). Data are mean ± SEM. *P < 0.05 and **P < 0.01. Statistical analysis was performed using Mann-Whitney U test with Benjamini-Hochberg false discovery rate correction.

**Fig. S4
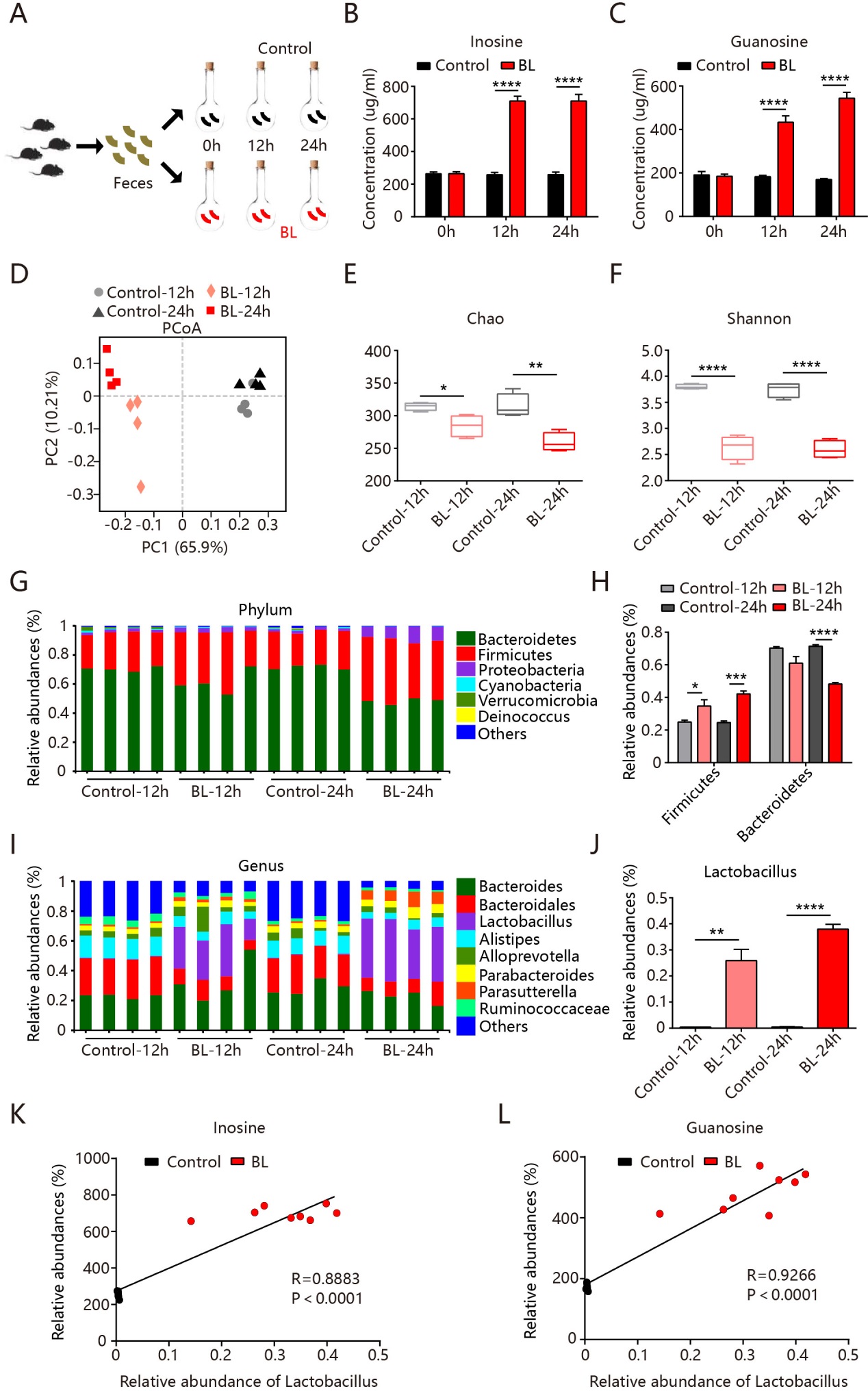
**

**Fig. S4 Barley leaf (BL) fermentation alters the gut microbiota composition and mediates enrichment of purine metabolites.** Fresh mice feces were co-cultured with or without BL under anaerobic conditions for 12 and 24 h. (A) Study design of in vitro anaerobic fermentation experiment. (B and C) Concentrations of inosine and guanosine in anaerobic cultures at 12 and 24 h of fermentation (n = 4). (D) Weighted UniFrac Principal coordinate analysis (PCoA) plot of the bacterial composition at the operational taxonomic unit (OTU) level (n = 4). (E and F) Alpha diversity analysis of gut bacterial richness (Chao1 index) and diversity (Shannon index) (n = 4). (G) Taxonomic distributions of bacterial composition at phylum level and (H) the relative abundances of Firmicutes and Bacteroidetes (n = 4). (I) Taxonomic distributions of bacterial composition at genus level and (J) the relative abundances of *Lactobacillus* (n = 4). (K and L) Pearson correlation analyses of the relative abundances of *Lactobacillus* and the concentrations of inosine and guanosine in anaerobic cultures. Data are mean ± SEM. *P < 0.05, **P < 0.01 and ****P < 0.0001. For analyzing relative abundance of the gut microbiota, two-tailed Wilcoxon rank-sum test by R Project were performed and the rest of the statistics was performed with Student’s *t* test.

**Fig. S5**

**
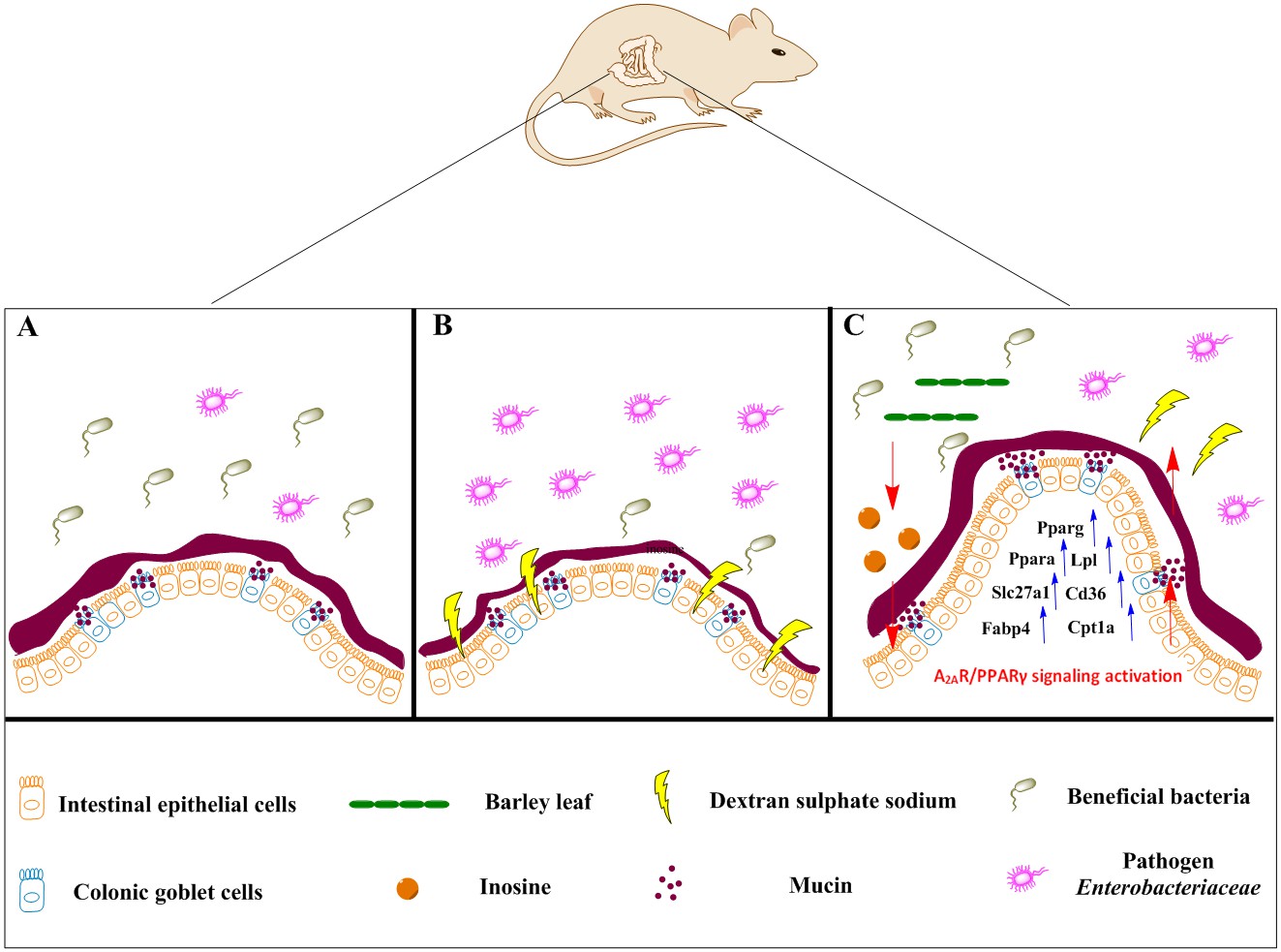
**

**Fig. S5 Proposed model for the preventive effects of barley leaf (BL) against dextran sulfate sodium (DSS)-induced colitis.** (A) Under steady-state conditions, colonic epithelial goblet cells produce and secrete mucins into the lumen to form mucus layer, which functions as a key barrier by separating the host epithelium from the gut microbiota. (B) In response to DSS challenge, mucosal barrier function is compromised, which is companied by dysbiosis of the gut microbiota characterized by the expansion of harmful bacteria such as *Enterobacteriaceae*. (C) Dietary supplementation of BL results in enrichment of microbiota-derived purine metabolite inosine, which can induce adenosine 2A receptor (A_2A_R)/peroxisome proliferator-activated receptor (PPAR)γ signaling activation in colonic tissues and reproduce the protective effects of BL against DSS-induced colitis through enhancing intestinal motility and improving mucosal barrier functions.

1. ***Correspondence:** College of Food Science and Nutritional Engineering, China Agricultural University, No.17, QinghuaEast Road, Haidian District, Beijing100083, China.

   **E-mail:** [chenfangch@sina.com](mailto:chenfangch@sina.com); **Tel/Fax:** +86-10-62737654 ext 18. [↑](#footnote-ref-1)
