# Supplementary material for: Gut microbiota-derived inosine from dietary barley leaf supplementation attenuates colitis through PPARγ signaling activation
Source: Microbiome. 2021 Apr 5;9:83. doi: 10.1186/s40168-021-01028-7 (PMC8022418; doi:10.1186/s40168-021-01028-7)
Supplement: Supplementary file 3 — Additional file 2: Table S1. The macronutrient composition of the BL powder in the present study. Table S2. The composition of CD and BL diet. Table S3. List of primers used in this study. [file 40168_2021_1028_MOESM3_ESM.docx]

**Additional file 2**

**Gut microbiota-derived inosine from dietary barley leaf supplementation attenuates colitis through PPARγ signaling activation**

Daotong Li, Yu Feng, Meiling Tian, Junfu Ji, Xiaosong Hu, Fang Chen^[[1]](#footnote-1)^*

**This file includes:**

Table S1. The macronutrient composition of the BL powder in the present study.

Table S2. The composition of CD and BL diet.

Table S3. List of primers used in this study.

**Table S1.** The macronutrient composition of the BL powder in the present study.

|  | **Unit (g/100g)** |
| --- | --- |
| Protein | 23.9 |
| Fat | 2.98 |
| Carbohydrate | 62.3 |
| Soluble dietary fiber | < 0.01 |
| Insoluble dietary fiber | 53.6 |
| Moisture | 3.99 |
| Ash | 6.8 |

**Table S2.** The composition of CD and BL diet.

| **Ingredients** **(g/kg of diet)** | **Diets** | |
| --- | --- | --- |
|  | **CD** | **BL** |
| **Barley leaf powder** | 0 | 25 |
| **Casein** | 189.58 | 178.58 |
| **L-Cysteine** | 2.84 | 2.84 |
| **Corn Starch** | 298.59 | 280.59 |
| **Maltodextrin** | 33.18 | 33.18 |
| **Sucrose** | 331.77 | 327.77 |
| **Cellulose** | 47.40 | 47.40 |
| **Soybean oil** | 23.70 | 23.70 |
| **Lard** | 18.96 | 18.96 |
| **Mineral Mix M1002** | 9.48 | 9.48 |
| **DiCalcium Phosphate** | 12.32 | 12.32 |
| **Calcium Carbonate** | 5.21 | 5.21 |
| **Potassium Citrate** | 15.64 | 15.64 |
| **Vitamin mix V10001** | 9.48 | 9.48 |
| **Choline Bitartrate** | 1.90 | 1.90 |
| **Total** | 1000 | 1000 |

All diets are isocaloric and contained 19.2% protein, 4.3% fat and 67.3% carbohydrate.

**Table 3**. List of primers used in this study.

| **Genes** | **Primers** | **Primer Sequences (5′-3′)** |
| --- | --- | --- |
| **Primers for qPCR** |  |  |
| *Mouse-Zo1* | F | 5’-AGTTCTGCCCTCAGCTACCA-3’ |
|  | R | 5’-GCTTAAAGCTGGCAGTGTC-3’ |
| *Mouse-Occludin* | F | 5’-ACAAAGAGCTCTCTCGTCTCG-3’ |
|  | R | 5’-CATAGTCTCCCACCATCCTC-3’ |
| *Mouse-Claudin2* | F | 5’-GTCAGCTTGCCAGAGACACT-3’ |
|  | R | 5’-TTCGCTTGTCTTTTGGCTGC-3’ |
| *Mouse-Claudin4* | F | 5’-GGGGATCATCCTGAGTTGTG-3’ |
|  | R | 5’-CACTGCATCTGACCTGTGCT-3’ |
| *Mouse-Reg3b* | F | 5’-CCCAGGCTTATGGCTCCTAC-3’ |
|  | R | 5’-ATGGAGCCCAATCCAAGTGT-3’ |
| *Mouse-Reg3g* | F | 5’-CGTGCCTATGGCTCCTATTGCT-3’ |
|  | R | 5’-TTCAGCGCCACTGAGCACAGAC-3’ |
| *Mouse-Defensin4* | F | 5’-TGGCCTCCAAAGGAGATAGACA-3’ |
|  | R | 5’-AGGCTGATCCTATCCAAAACACA-3’ |
| *Mouse-Mmp7* | F | 5’-GGCTTCGCAAGGAGAGATCA-3’ |
|  | R | 5’-GCCAAATTCATGGGTGGCAG-3’ |
| *Mouse-Lpl* | F | 5’-GCAGCTCCGGTGATATAGAGG-3’ |
|  | R | 5’-AAGGTCATCTTCTGTGCTAGG-3’ |
| *Mouse-Scd1* | F | 5’-CCGGAGACCCCTTAGATCGA-3’ |
|  | R | 5’-TAGCCTGTAAAAGATTTCTGCAAACC-3’ |
| *Mouse-Ppara* | F | 5’-TACTGCCGTTTTCACAAGTGC-3’ |
|  | R | 5’-AGGTCGTGTTCACAGGTAAGA-3’ |
| *Mouse-Pparg* | F | 5’-GGAAGACCACTCGCATTCCTT-3’ |
|  | R | 5’-GTAATCAGCAACCATTGGGTCA-3’ |
| *Mouse-Cd36* | F | 5’-GGAACTGTGGGCTCATTGC-3’ |
|  | R | 5’-CATGAGAATGCCTCCAAACAC-3’ |
| *Mouse-Fabp4* | F | 5’-TGGGAACCTGGAAGCTTGTCTC-3’ |
|  | R | 5’-GAATTCCACGCCCAGTTTGA-3’ |
| *Mouse-Slc27a1* | F | 5’-CATGTGTACCCCATCCGTCT-3’ |
|  | R | 5’-CTGTGGGCAATCTTCTTGTTG-3’ |
| *Mouse-Cpt1a* | F | 5’-AAAGATCAATCGGACCCTAGACA-3’ |
|  | R | 5’-CAGCGAGTAGCGCATAGTCA-3’ |
| *Mouse-Gapdh* | F | 5’-GTGTTCCTACCCCCAATGTGT-3’ |
|  | R | 5’-ATTGTCATACCAGGAAATGAGCTT-3’ |
| *Human-Lpl* | F | 5’-CGGATTAACATTGGAGAAGCTATCCG-3’ |
|  | R | 5’-AGCTGGTCCACATCTCCAAGTC-3’ |
| *Human-Scd1* | F | 5’-CAGTGCAATGCTTGTAGAAGTAGG-3’ |
|  | R | 5’-GGAATGCTGGTTAGTTTGCTGA-3’ |
| *Human-Cd36* | F | 5’-CGATTAACATAAGTAAAGTTGCCATATCG-3’ |
|  | R | 5’-CGCAGTGACTTTCCCAATAGGAC-3’ |
| *Human-Slc27a1* | F | 5’-TGCCGAGAGTGGAACACAC-3’ |
|  | R | 5’-AAAAGCAGCTGGACCCTACA-3’ |
| *Human-Fabp4* | F | 5’-TGGATTCGACTTAGACTTGACCT-3’ |
|  | R | 5’-GGTGGGTTATGGTCTTCAAAAGG-3’ |
| *Human-Cpt1a* | F | 5’-TCCAGTTGGCTTATCGTGGTG-3’ |
|  | R | 5’-TCCAGAGTCCGATTGATTTTTGC-3’ |
| *Human-Pparg* | F | 5’-GAGGAGCCTAAGGTAAGGAG-3’ |
|  | R | 5’-GTCATTTCGTTAAAGGCTGA-3’ |
| *Human-Gapdh* | F | 5’-CGGACCAATACGACCAAATCCG-3’ |
|  | R | 5’-AGCCACATCGCTCAGACACC-3’ |
| **Primer for sequencing** | 338F | 5’-GTGCCAGCMGCCGCGG-3’ |
|  | 806R | 5’-CCGTCAATTCMTTTRAGTTT-3’ |
| **Sequence for siRNA** | PPARγ | GAACAUCGAGUGUCGAAUATT |
|  | A_2A_R | UGCUCAUGCUGGGUGUCUAUU |

1. ***Correspondence:** College of Food Science and Nutritional Engineering, China Agricultural University, No.17, QinghuaEast Road, Haidian District, Beijing100083, China.

   **E-mail:** [chenfangch@sina.com](mailto:chenfangch@sina.com); **Tel/Fax:** +86-10-62737654 ext 18. [↑](#footnote-ref-1)
